# Optimisation and mechanistic characterization of a selective benzoxazine-based MAP4K4 inhibitor in pancreatic cancer cells

**DOI:** 10.1080/14756366.2026.2743434

**Published:** 2026-10-16

**Authors:** Yi-Wen Wu, Chun-Lin Yang, Yu-Ting Fang-Chin, Yun-Hsuan Yeh, Bor-Jiun Chen, Tzu-Ying Sung, Jung-Chun Chu, Tony Eight Lin, Shih-Chung Yen, Jui-Hua Hsieh, Sing-Han Huang, Cheng-Ying Chu, Kai-Chien Yang, Kai-Cheng Hsu, Shiow-Lin Pan

**Affiliations:** ^a^ Graduate Institute of Cancer Biology and Drug Discovery, College of Medical Science and Technology, Taipei Medical University, Taipei, Taiwan; ^b^ Ph.D. Program for Cancer Molecular Biology and Drug Discovery, College of Medical Science and Technology, Taipei Medical University, Taipei, Taiwan; ^c^ Warshel Institute for Computational Biology, School of Medicine, The Chinese University of Hong Kong (Shenzhen), Shenzhen, Guangdong, China; ^d^ Division of Translational Toxicology, National Institute of Environmental Health Sciences, National Institutes of Health, Durham, NC, USA; ^e^ Department of Digital Biology, Graphen Drugomics Inc., New York, NY, USA; ^f^ CRISPR Gene Targeting Core, Taipei Medical University, Taipei, Taiwan; ^g^ TMU Research Center of Cancer Translational Medicine, Taipei Medical University, Taipei, Taiwan; ^h^ Department and Graduate Institute of Pharmacology, National Taiwan University College of Medicine, Taipei, Taiwan; ^i^ Ph.D. Program in Innovative Medical Technology Research and Development Industry, College of Medical Science and Technology, Taipei Medical University, Taipei, Taiwan; ^j^ Cancer Center, Wan Fang Hospital, Taipei Medical University, Taipei, Taiwan

**Keywords:** MAP4K4, benzoxazine-based inhibitor, structure-activity relationship, pancreatic cancer

## Abstract

Pancreatic ductal adenocarcinoma (PDAC) remains a lethal malignancy with limited targeted therapeutic options. Mitogen-activated protein kinase kinase kinase kinase 4 (MAP4K4) has been implicated in PDAC progression, but the development of effective MAP4K4 inhibitors requires further improvement in potency, selectivity, and mechanistic characterisation. We previously identified F389-0746 as a selective benzoxazine-based MAP4K4 inhibitor with anti-cancer activity in pancreatic cancer. Here, we used the F389-0746 scaffold to guide the selection and evaluation of benzoxazine-based analogs, followed by biochemical, structural, selectivity, and cellular characterisation of the most potent analogue. Among the identified analogs, F389-0736 showed the strongest MAP4K4 inhibitory activity, with an IC_50_ value of 44.0 nM, representing an approximately threefold improvement over F389-0746. Docking and structure-activity relationship analyses identified key binding interactions that support the enhanced potency of F389-0736 within the MAP4K4 binding pocket. Kinase profiling further demonstrated the selectivity of F389-0736 for MAP4K4 across the tested kinase panel. CETSA further demonstrated F389-0736-induced thermal stabilisation of MAP4K4, supporting intracellular target engagement. In pancreatic cancer cells, F389-0736 suppressed TAK1, JNK, and p38 phosphorylation and significantly reduced cell viability and proliferation, with IC_50_ and GI_50_ values of 4.41–4.65 μM and 4.03–5.12 μM, respectively. F389-0736 also inhibited clonogenic growth and induced sub-G1 accumulation with activation of apoptotic signalling. RNA sequencing identified 398 differentially expressed genes and revealed enrichment of inflammatory and stress response pathways, programmed cell death programs, oncogenic signalling perturbation, and metabolic reprogramming. These findings identify F389-0736 as a potent and selective benzoxazine-based MAP4K4 inhibitor and support its use as a chemical probe and lead scaffold for investigating MAP4K4-dependent signalling and therapeutic vulnerability in pancreatic cancer.

## Introduction

Pancreatic ductal adenocarcinoma (PDAC) represents one of the most lethal malignancies, ranking as the third leading cause of cancer-related deaths in the United States and expected to become the second leading cause in the near future.^1–3^ The disease is characterised by delayed diagnosis and limited targeted therapeutic options. Current treatment relies on systemic chemotherapy, with FOLFIRINOX and gemcitabine plus nab-paclitaxel as the standard regimens.^4^ However, the five-year survival rate remains approximately 13%, showing minimal improvement compared to other cancer types.^5^ This poor prognosis persists due to limited tolerance of aggressive multi-agent chemotherapy regimens and insufficient targeted therapeutic options. Despite recent approval of targeted therapies for BRCA mutations, MSI-H, and NTRK fusions, these precision medicine strategies are available to fewer than 10% of patients.^4^ This narrow therapeutic coverage underscores the urgent need to identify alternative kinase targets and develop novel targeted therapies for PDAC patients.

Mitogen-activated protein kinase kinase kinase kinase 4 (MAP4K4), also known as HGK, has emerged as a promising therapeutic target for PDAC. MAP4K4 is overexpressed in pancreatic cancer tissues and cell lines. The high expression levels are directly correlated with increased tumour size, advanced disease stage, and lymph node metastasis.^6–9^ MAP4K4 promotes pancreatic cancer cell progression by phosphorylating MLK3, thereby activating downstream JNK signalling and driving cell proliferation, invasion, and metastasis.^6^^,^^9^^,^^10^ Genetic knockdown or pharmacologic inhibition of MAP4K4 suppresses cell proliferation, colony formation, and migration in PDAC cell lines, while inducing G1 phase cell cycle arrest and apoptosis.^10–16^ These genetic and pharmacological studies validate MAP4K4 as a critical driver of PDAC progression and a promising therapeutic target for pancreatic cancer.

Although MAP4K4 represents a validated therapeutic target, no inhibitors have yet advanced to clinical evaluation for cancer. Several compounds with submicromolar potency have been developed in preclinical studies; however, inadequate kinase selectivity has prevented their clinical translation. For example, the isoquinoline-based inhibitor GNE-495 displays activity against MINK1 and MLK1,^17^ the aminopyridine-based compound PF-06745013 inhibits TRKC and FLT3,^18^ and the pyrrolotriazine compound CHEMBL3326802 inhibits MINK1 and FLT3.^19^ Off-target MINK1 inhibition may reduce anti-tumour efficacy by stabilising YAP in the Hippo pathway,^20^^,^^21^ while FLT3 inhibition could compromise anti-tumour immunity through dendritic cell depletion.^22^ This lack of selectivity underscores the critical need for developing highly selective MAP4K4 inhibitors for pancreatic cancer treatment.

We previously developed a benzoxazine-based MAP4K4 inhibitor, F389-0746, using structure-based virtual screening.^9^ This compound demonstrated high selectivity with an IC_50_ value of 120.7 nM. F389-0746 effectively suppressed JNK signalling by inhibiting phosphorylation of downstream effectors (MKK4, JNK, and c-Jun), thereby reducing pancreatic cancer cell growth and inducing apoptosis *in vitro*. Consistently, in vivo xenograft studies demonstrated tumour growth inhibition comparable to gemcitabine treatment.^9^ Despite these promising results, there remained scope for further potency enhancement to support lead development. Structure-activity relationship (SAR) analysis from our previous study provided opportunities for rational structural optimisation. Additionally, while MAP4K4 inhibition clearly impacts JNK signalling, the broader downstream mechanisms remain incompletely characterised. The benzoxazine-based scaffold provides an opportunity to develop chemical probes to investigate MAP4K4-related mechanisms by RNA sequencing (RNA-seq) analysis due to its high kinome selectivity.

In this study, we aimed to develop more potent benzoxazine-based MAP4K4 inhibitors with selectivity for pancreatic cancer therapy. The workflow of this study is illustrated in Figure 1. First, analogs were identified from the ChemDiv compound library and evaluated for their MAP4K4 inhibitory activity using enzymatic assays. SAR analysis was then conducted to identify key structural features that modulate inhibitory potency. The most potent inhibitor was subsequently assessed for its effects on pancreatic cancer cell viability and proliferation *in vitro*. The compound was further evaluated for its ability to suppress MAP4K4-mediated signalling pathways, alter cell cycle distribution, and induce apoptosis. Additionally, compound selectivity was evaluated by testing against a panel of kinases representing diverse families across the kinome. Finally, RNA-seq analysis was performed to elucidate the transcriptomic changes and biological pathways modulated by MAP4K4 inhibition. In summary, this study identified a nanomolar potent benzoxazine analogue with high selectivity, which also serves as a useful chemical probe to further elucidate the biological functions and therapeutic potential of MAP4K4 in pancreatic cancer.

**Figure 1.**
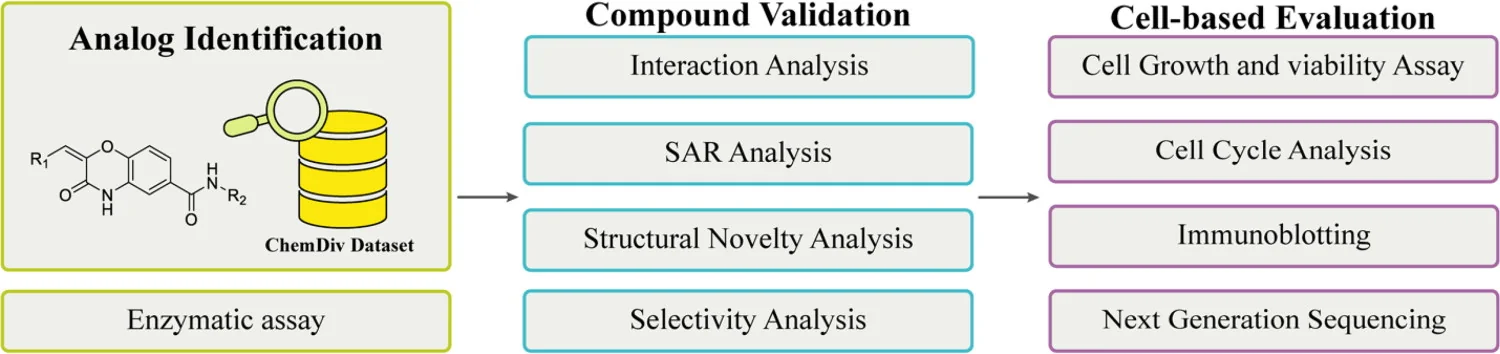
Workflow of the study. Benzoxazine-based analogs were identified from the ChemDiv database and evaluated for MAP4K4 inhibition. The structural and interaction features associated with inhibitory potency were further analysed. The effects and mechanisms of the selected inhibitor on cancer cells were further investigated.

## Methods

### Molecular docking

The crystal structure of MAP4K4 (PDB ID: 4OBP) was obtained from the RCSB Protein Data Bank.^23^ Molecular docking was performed using LeadIT.^24^ The protein structure was prepared, and water molecules were removed from the binding site before docking. The binding site was centred on the co-crystallized ligand (HET ID: 2QU) and defined as all residues within a radius of 12 Å. Analogs were identified from the ChemDiv compound library, which contains approximately 1.6 million compounds. The identified analogs were protonated under aqueous conditions and docked into the MAP4K4 binding site using the default LeadIT settings. Protein-compound interactions were subsequently analysed using Pipeline Pilot.^25^

### Kinase assay

Kinase inhibitory activity was measured using a fluorescence resonance energy transfer (FRET)-based Z′LYTE biochemical assay (Thermo Fisher Scientific). The assay uses a synthetic peptide substrate labelled with donor and acceptor fluorophores. During the kinase reaction, the kinase transfers the γ-phosphate of ATP to a single tyrosine, serine, or threonine residue in the FRET peptide. A site-specific protease is subsequently added to cleave non-phosphorylated peptides, whereas phosphorylated peptides are resistant to cleavage. Proteolytic cleavage disrupts FRET between the donor and acceptor fluorophores, while uncleaved phosphorylated peptides maintain FRET. Reaction progress was quantified using the ratio of donor emission to acceptor emission following excitation of the donor fluorophore at 400 nm. A higher emission ratio indicates reduced peptide phosphorylation and greater kinase inhibition. The selected compounds were evaluated at the indicated concentrations, and half-maximal inhibitory concentrations (IC_50_) were calculated using GraphPad Prism. Each kinase assay was performed in duplicate according to Thermo Fisher Scientific’s quality-control guidelines. Kinase selectivity profiling was conducted using Thermo Fisher Scientific’s SelectScreen^®^ Kinase Profiling Service. Z′-LYTE biochemical assays were used according to the assay format validated for each kinase. Detailed assay protocols are available from Thermo Fisher Scientific (Z′-LYTE: www.thermofisher.com/z-lyte). The selectivity of the compound was evaluated using the Eurofins kinase profiling service.

### Cell culture

Human pancreatic cancer cell lines Mia PaCa-2 and Panc-1 were obtained from the Bioresource Collection and Research Centre (BCRC, Hsinchu, Taiwan). Cells were maintained in Dulbecco’s modified Eagle’s medium (DMEM) containing 10% foetal bovine serum, 100 units/mL penicillin, and 100 μg/mL streptomycin. All cells were cultured at 37 °C in a humidified atmosphere containing 5% CO_2_.

### Cell viability analysis

Cell viability was determined using the MTT assay. Mia PaCa-2 and Panc-1 cells were seeded into 96-well plates at densities of 3 × 10³ and 5 × 10³ cells per well, respectively. After cell attachment, Mia PaCa-2 and Panc-1 cells were exposed to F389-0736 at 0.1, 0.3, 1, 3, 10, and 30 μM for 72 h. For pharmacological comparison, GNE-495 and gemcitabine were included as reference compounds and evaluated at the indicated concentrations (0.1, 0.3, 1, 3, and 10 μM) under the same experimental conditions. MTT reagent was then added to each well and incubated for 1 h at 37 °C. The resulting formazan crystals were dissolved in DMSO, and absorbance was measured at 550 nm using a Synergy HTX microplate reader. Cell viability was calculated relative to the untreated control group.

### Cell proliferation analysis

Cell proliferation was assessed by sulforhodamine B (SRB) staining. Mia PaCa-2 and Panc-1 cells were seeded in 96-well plates at 3 × 10³ and 5 × 10³ cells per well, respectively. After overnight attachment, cells were treated with F389-0736 for 72 h. Mia PaCa-2 and Panc-1 cells were treated with 0.1, 0.3, 1, 3, 10, and 30 μM F389-0736. GNE-495 and gemcitabine were included as reference compounds and evaluated at the indicated concentrations under the same experimental conditions. Following treatment, cells were fixed with 10% trichloroacetic acid, washed with distilled water, and stained with 0.4% SRB solution. Excess dye was removed by washing with 1% acetic acid, and protein-bound dye was solubilised with 10 mM Tris-base. Absorbance was measured at 515 nm using a Synergy HTX microplate reader. Cell proliferation was expressed relative to the basal cell number measured at the time of treatment initiation. GI_50_ values were calculated as the concentrations that reduced net cell growth by 50% relative to untreated cells.

### Colony formation

The long-term effect of F389-0736 on clonogenic growth was evaluated using a colony formation assay. Mia PaCa-2 and Panc-1 cells were seeded in 6-well plates at 1 × 10³ cells per well and allowed to attach before treatment with F389-0736 at 0.1, 0.3, 1, 3, and 10 μM. Gemcitabine at 0.1 and 0.3 μM was included as a positive control for inhibition of clonogenic growth. Cells were cultured for two weeks, and the medium containing the indicated concentration of F389-0736 was refreshed periodically. At the end of the treatment period, colonies were fixed and stained with crystal violet. Colonies containing more than 50 cells were counted, and colony formation was expressed as a percentage of the untreated control group.

### Cellular thermal shift assay

Cellular thermal shift assay (CETSA) was performed by MedChemExpress to evaluate the intracellular target engagement of the compound with MAP4K4. Mia PaCa-2 cells were treated with 10 μM compound or vehicle control and subsequently divided into equal aliquots. The samples were heated at temperatures ranging from 45 °C to 59 °C at 2 °C intervals. After heat treatment, cells were lysed and centrifuged to remove heat-induced protein aggregates. The soluble protein fractions were collected and analysed by western blotting using an anti-MAP4K4 antibody. The thermal stability of MAP4K4 was assessed by comparing the remaining soluble MAP4K4 protein between F389-0736 treated and vehicle-control samples at each temperature.

### Western blot analysis

Mia PaCa-2 and Panc-1 cells were treated with F389-0736 at the indicated concentrations and time points. For signalling pathway analysis, cells were treated with 0.3, 1, 3, and 10 μM F389-0736 for 4 h. GNE-495 (3 μM) was included as a reference MAP4K4 inhibitor. For apoptosis-related protein analysis, cells were treated with 0.3, 1, 3, and 10 μM F389-0736 for 72 h, and gemcitabine (3 and 10 μM) was included as a positive control for apoptosis induction. After treatment, cells were lysed in RIPA buffer supplemented with protease and phosphatase inhibitors. Cell lysates were collected and centrifuged at 14,000 rpm for 15 min at 4 °C, and protein concentrations were determined using a BCA protein assay.

Equal amounts of protein were mixed with sample buffer, denatured at 95 °C, separated by SDS-PAGE, and transferred onto PVDF membranes. Membranes were blocked with 5% non-fat milk in TBST for 1 h at room temperature and then incubated with primary antibodies overnight at 4 °C. The analysed proteins included p-TAK1, p-JNK, total JNK, p-p38, total p38, Bcl-2, PARP, caspase-9, caspase-3, cleaved caspase-3, and GAPDH. After washing, membranes were incubated with HRP-conjugated secondary antibodies for 1 h at room temperature. Protein signals were detected using an enhanced chemiluminescence reagent. Relative protein levels were quantified using ImageJ software and normalised to the untreated control group.

### Cell cycle analysis

Cell cycle distribution was analysed by flow cytometry following propidium iodide staining. Mia PaCa-2 and Panc-1 cells were seeded into culture dishes (3 × 10^5^ cells/dish) and treated with F389-0736 at 0.3, 1, 3, and 10 μM for 72 h. After treatment, cells were harvested, washed with cold PBS, and fixed in 70% ethanol at −20 °C. Fixed cells were washed to remove ethanol and then stained with propidium iodide staining buffer containing PI, RNase A, and Triton X-100. DNA content was analysed using a BD Accuri^™^ Flow Cytometer, and the percentages of cells in the sub-G1, G0/G1, S, and G2/M phases were calculated using the accompanying analysis software (Becton Dickinson, Mountain View, CA, USA).

### RNA sequencing analysis

Mia PaCa-2 cells were exposed to 10 μM of the compound for 24 h, after which total RNA was isolated according to the Direct-zol RNA Kit protocol. The RNA purity and concentration were measured using SimpliNano-Biochrom Spectrophotometers (Biochrom, MA, USA). Sequencing libraries were generated from 1 μg of total RNA using the KAPA mRNA HyperPrep Kit (KAPA Biosystems, Roche, Switzerland). The quality of the libraries was evaluated using the Qubit 2.0 Fluorometer (Thermo Scientific) and the Agilent Bioanalyzer 2100 system. Finally, sequencing was performed on the Illumina NovaSeq 6000 platform. Raw read count data were analysed using the R package DESeq2 to identify differentially expressed genes (DEGs). Pathway annotations were derived from the KEGG database.^26^ Pathway enrichment was assessed using a hypergeometric test, and enrichment scores were subsequently standardised as z-scores.

### Statistical analysis

Cell-based biological experiments were independently repeated at least three times, unless otherwise specified in the corresponding figure legends. Data are expressed as the mean ± standard deviation (SD) or as percentages normalised to the untreated control group, as specified in the figure legends. For cell-based experiments involving comparisons among multiple treatment groups, differences were analysed using the Kruskal-Wallis test followed by Dunn’s multiple-comparison test. A *p* value below 0.05 was considered statistically significant. Statistical significance is denoted as *p < 0.05 and **p < 0.01 compared with the untreated control group. RNA-seq analysis was performed using two independent biological replicates per group (*n* = 2). The number of biological replicates and the statistical analysis used for each experiment are specified in the corresponding figure legends.

## Results

### Identification and evaluation of benzoxazine-based MAP4K4 analogs

To identify more potent benzoxazine-based inhibitors, analogs of F389-0746 were searched from the ChemDiv database, which contains approximately 1.6 million compounds. As illustrated in Figure 2(A), F389-0746 can be divided into four structural groups. Previous interaction analysis revealed that Group 2 forms a critical hydrogen bond with residue D115, while Group 3 mimics an adenine moiety and engages hinge residues, suggesting their essential roles in MAP4K4 inhibition. Therefore, compounds retaining Groups 2 and 3 of F389-0746 were selected, as they were expected to adopt similar binding modes (Figure 2(B)).

**Figure 2.**
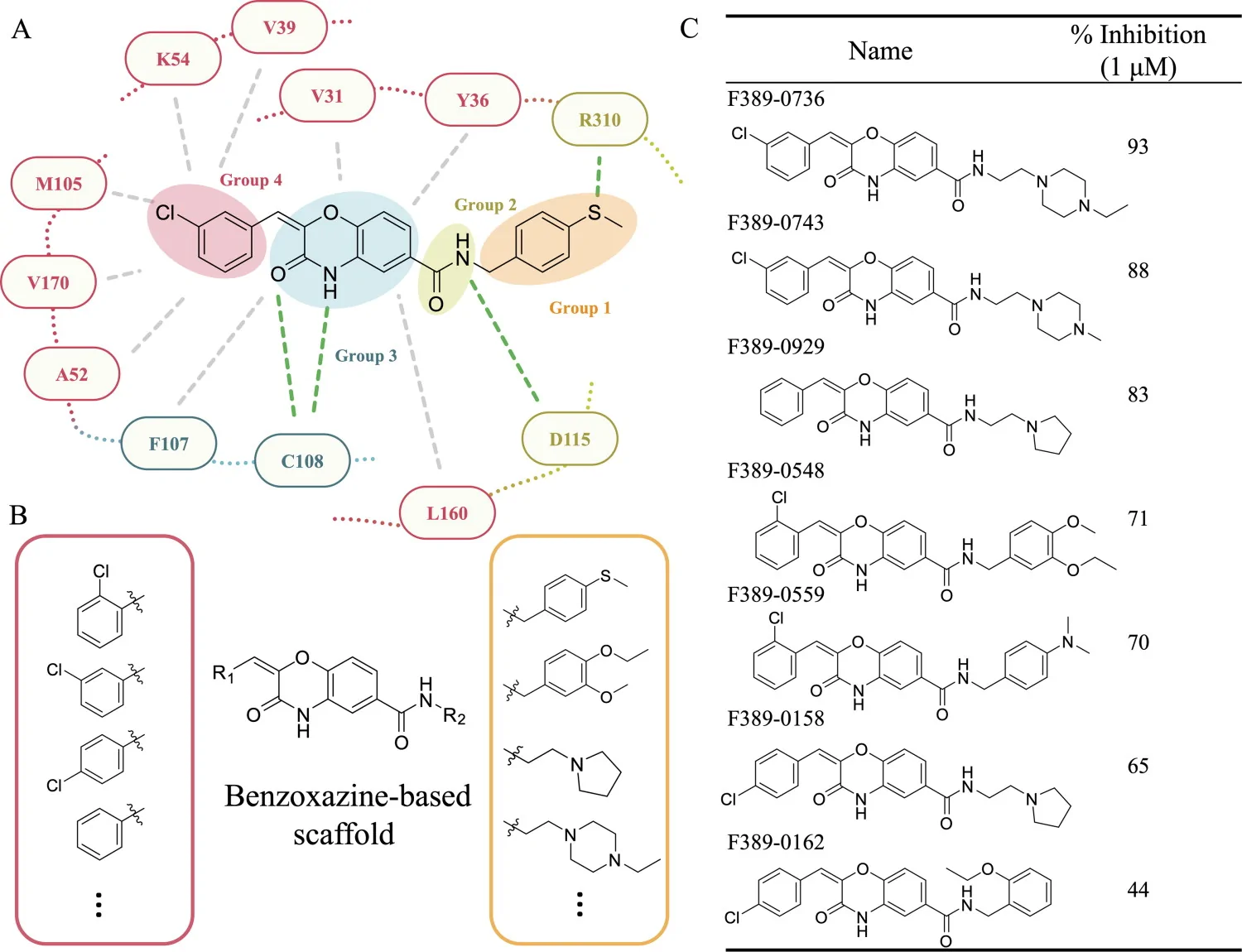
Identification and inhibitory profiling of benzoxazine-based analogs. (A) A two-dimensional interaction diagram illustrating the interactions between F389-0746 and MAP4K4, in which exterior residues (yellow) interact with Groups 1 and 2, hinge residues (blue) interact with Group 3, and interior residues (red) interact with Group 3 and 4. Hydrogen bonds are represented as green dashed lines, and hydrophobic interactions are represented as gray dashed lines. (B) A schematic diagram of the benzoxazine-based scaffold highlighting the conserved structural features retained for analogue selection. (C) Inhibitory activity of seven identified benzoxazine-based analogs against MAP4K4 at 1 μM.

Seven analogs were identified and evaluated for their inhibitory activity against MAP4K4. Among them, six analogs demonstrated more than 50% inhibition at 1 μM concentration (Figure 2(C)). Based on their inhibitory activity, the three most potent analogs, F389-0736, F389-0743, and F389-0929, were selected for IC_50_ determination. Notably, F389-0736 and F389-0743 exhibited IC_50_ values below 100 nM. Among these analogs, F389-0736 showed the highest potency, with an IC_50_ value of 44.0 nM, representing a threefold improvement over F389-0746 (IC_50_ = 120.7 nM) (Table 1 and Supplementary Figure 1). These results confirm that benzoxazine-based inhibitors retaining Groups 2 and 3 maintain high inhibitory potency. Moreover, these findings validate the SAR insights and demonstrate their utility for guiding rational optimisation of MAP4K4 inhibitors.

**Table 1.** IC_50_ values of identified analogs.

Name	IC_50_ (nM)
F389-0736 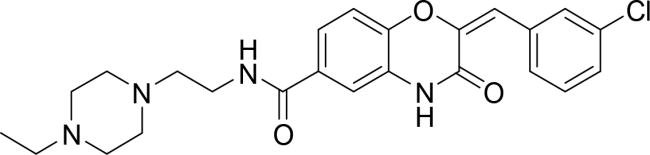	44.0
F389-0743 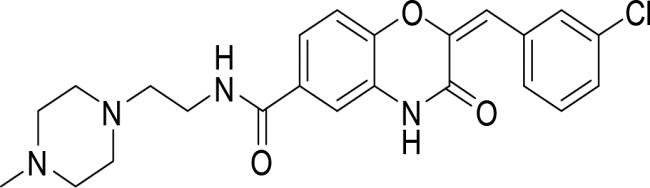	86.9
F389-0929 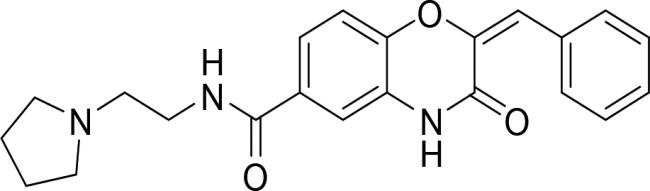	184.7

### Interaction analysis of F389-0736

To investigate the molecular interactions between compound F389-0736 and MAP4K4, molecular docking was conducted to analyse its binding conformation. As illustrated in Figure 3, the compound can be divided into four structural groups, designated as Groups 1–4. Group 1 consists of a basic piperazine substituent located at the exterior of the binding site, forming two hydrogen bonds with the carboxylate of residue D115. Group 2 serves as a linker, with the nitrogen atom acting as a hydrogen bond donor to form an additional hydrogen bond with D115. Group 3 contains a benzoxazine-based scaffold that mimics an adenine moiety and serves as the hinge-binding core of F389-0736. Docking results reveal that this scaffold forms two hydrogen bonds with the backbone of hinge residue C108. Moreover, it is surrounded by residues V31, F107, and L160, thereby forming multiple hydrophobic interactions. Group 4 comprises a phenyl ring positioned in the interior of the binding site. This ring establishes a π-π interaction with the side chain of Y36 and engages in hydrophobic interactions with residues V39, A52, K54, M105, and V170. Collectively, these observations indicate that F389-0736 occupies the MAP4K4 binding site through hydrogen-bonding and hydrophobic interactions, thereby inhibiting MAP4K4 kinase activity.

**Figure 3.**
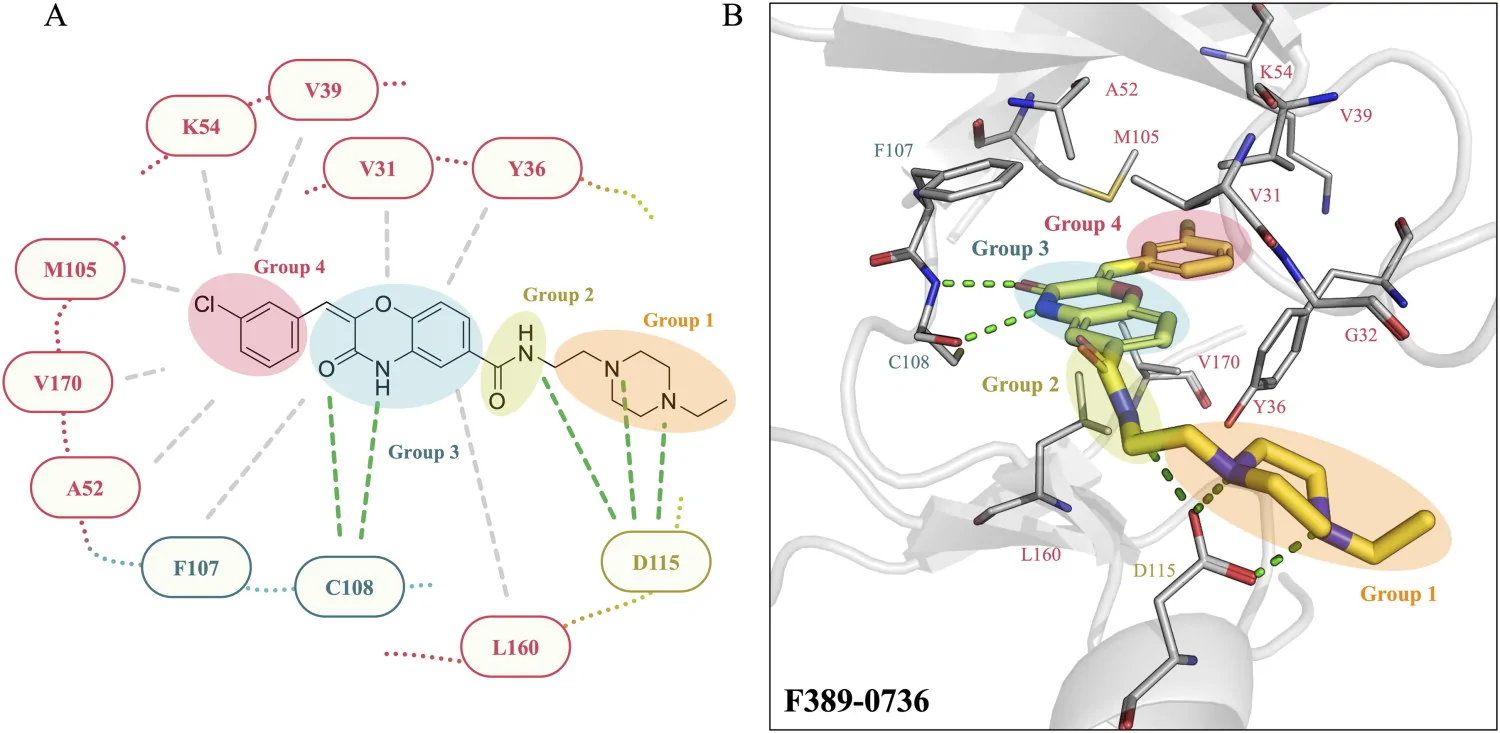
Docking pose and interactions of compound F389-0736 in the MAP4K4 binding site. (A) The 2D interaction figure shows exterior residues (yellow) interact with Groups 1 and 2, hinge residues (blue) interact with Group 3, and interior residues (pink) interact with Group 3 and 4. Hydrogen bonds are represented as green dashed lines, and hydrophobic interactions are represented as gray dashed lines. (B) Compound F389-0736 (yellow) shows favourable occupation of the MAP4K4-binding site. Residues from the interior, exterior, and hinge regions are labelled and coloured red, yellow, and blue, respectively. The compound structures are segmented into four regions: Group 1 (orange), Group 2 (lime), Group 3 (blue), and Group 4 (red).

### SAR analysis

To investigate the relationship between compound structures and inhibitory activity, we analysed a panel of 28 analogs, including compounds spanning high (>65%), moderate (30–65%), and low (<30%) inhibitory activity at 1 μM.^9^ All compounds maintained conserved hinge-binding interactions, while structural variations at the R_1_ and R_2_ positions altered their engagement with the interior and exterior residues and consequently modulated inhibitory activity (Table 2). Modifications at the R_2_ position strongly influenced inhibitory activity. The most potent analogs, F389-0736 and F389-0743, both contained extended nitrogen-containing heterocycles capable of forming salt bridges with the D115 carboxylate. F389-0736, in particular, exhibited approximately threefold higher potency than F389-0746 due to its piperazine substituent establishing dual salt bridges with D115, whereas the thiophenol moiety in F389-0746 formed only a single hydrogen bond with R310 (Figure 2(A)). These results indicate that compounds forming salt bridges with D115 confer superior inhibitory activity compared to those forming hydrogen bonds with R310. Consistent with this trend, methoxy and ethoxy substituents at the R_2_ position exhibited reduced potency by preferentially engaging R310 rather than D115. For instance, F389-0944 formed hydrogen bonds with R310 and achieved only 40% inhibition at 1 μM (Figure 4(B)). Compounds lacking hydrophilic R_2_ substituents could not engage exterior residues and displayed poor activity. For example, F389-0577 and F389-0011, which lacked hydrophilic R_2_ substituents, exhibited only 27% and 19% inhibition at 1 μM, respectively.

**Figure 4.**
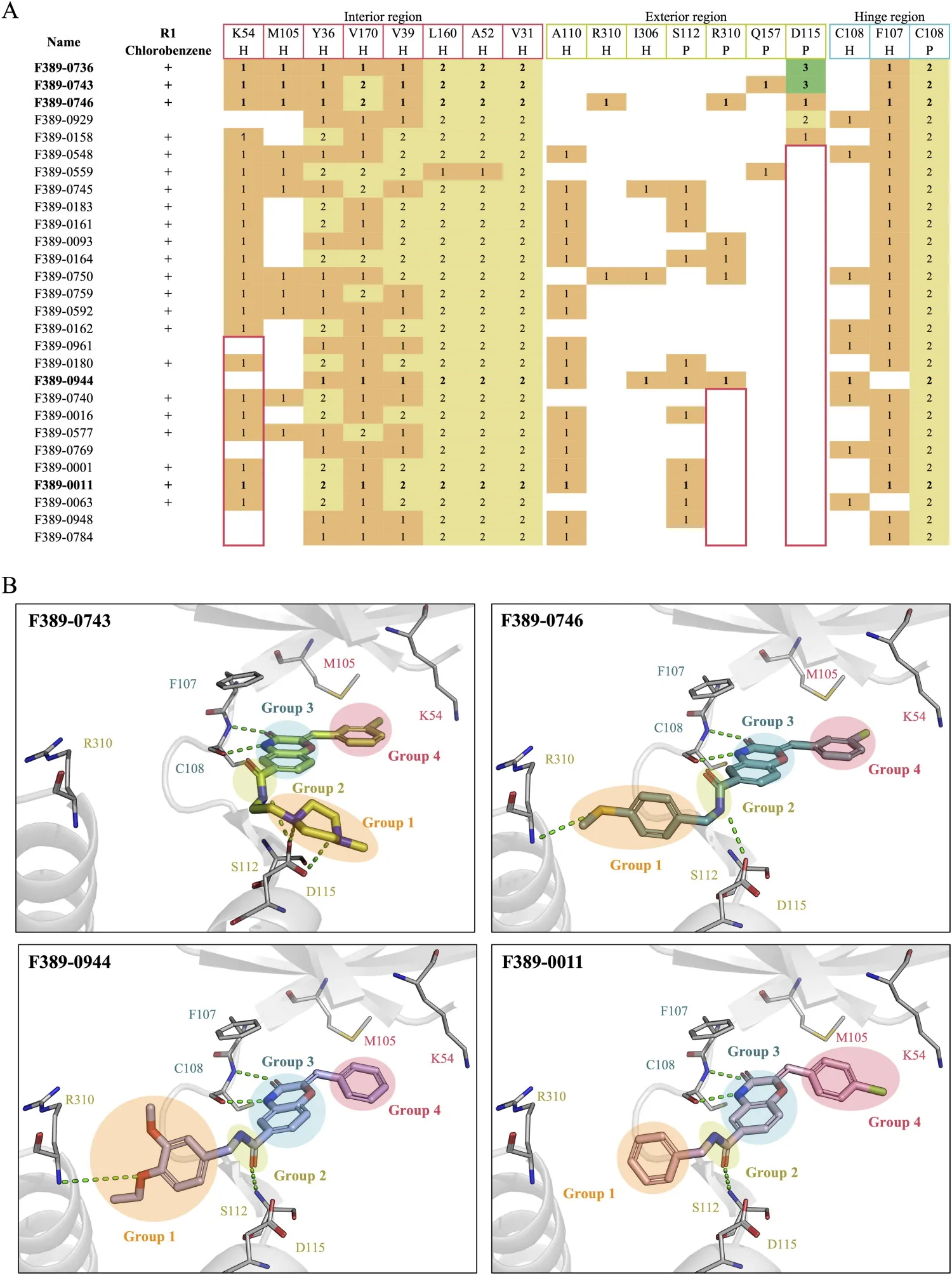
SAR analysis of analogs. (A) Interaction profiles of the analogs. Residues in the MAP4K4 binding site are categorised into interior, exterior, and hinge regions. H and P denote hydrophobic and polar contacts, respectively. Polar contacts include salt bridges and hydrogen bonding. The number of interactions formed by each compound is represented by a colour scale: brown (one), yellow (two), and green (three). (B) Docked conformations of the analogs showing the key interactions within the MAP4K4 binding site. Hydrogen bonds are shown as green dashed lines. Residues from the interior, exterior, and hinge regions are labelled and coloured red, yellow, and blue, respectively. The compound structures are segmented into four regions: Group 1 (orange), Group 2 (lime), Group 3 (blue), and Group 4 (red).

**Table 2.** Inhibitory activity of F389-0736 analogs.

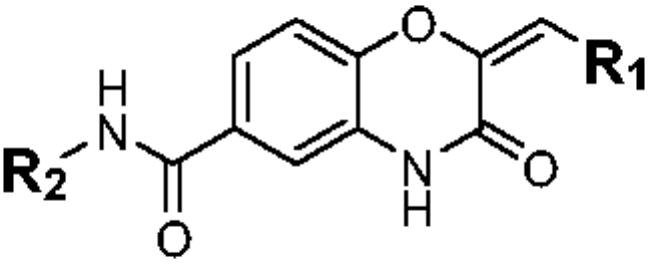											
Compound	R_1_	R_2_	% Inhibition (1 μM)	Compound	R_1_	R_2_	% Inhibition (1 μM)	Compound	R_1_	R_2_	% Inhibition (1 μM)
F389-0736	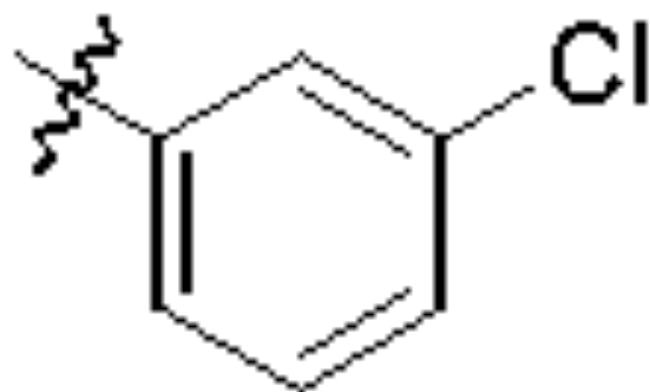	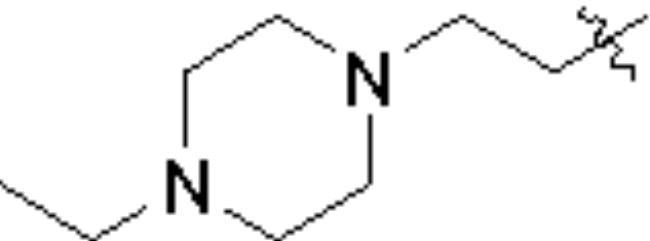	93	F389-0559	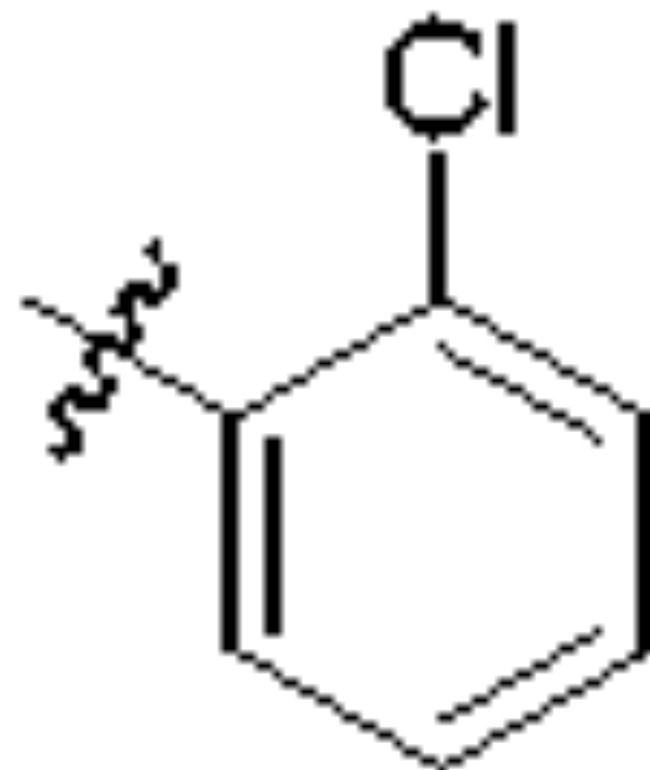	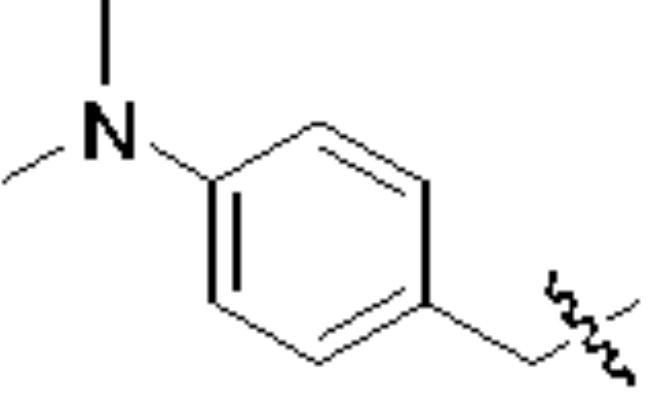	70	F389-0944	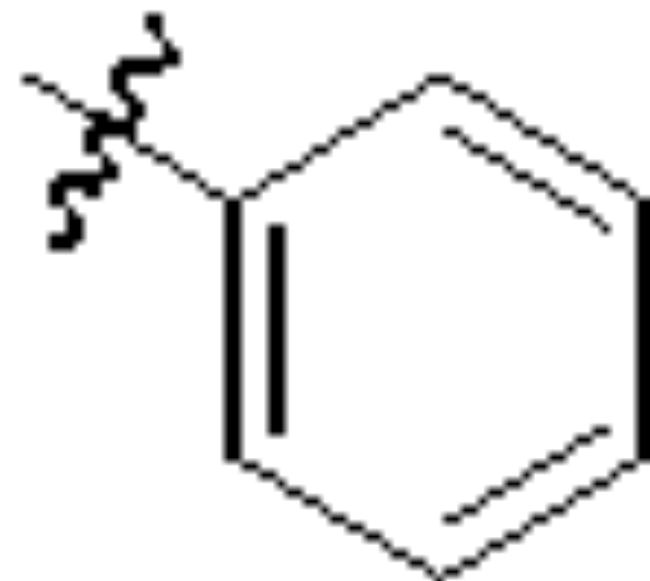	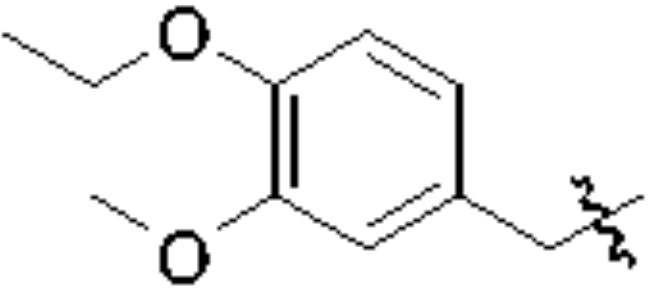	40
F389-0743	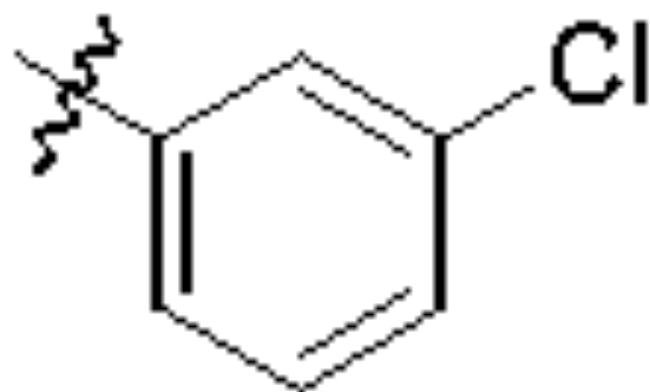	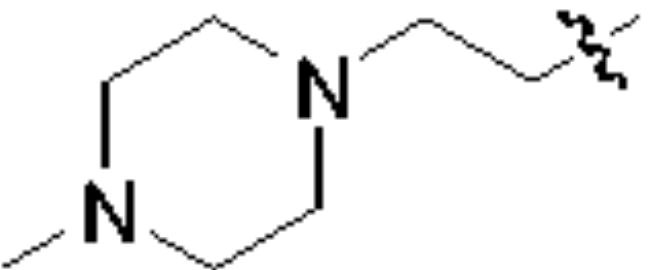	88	F389-0164	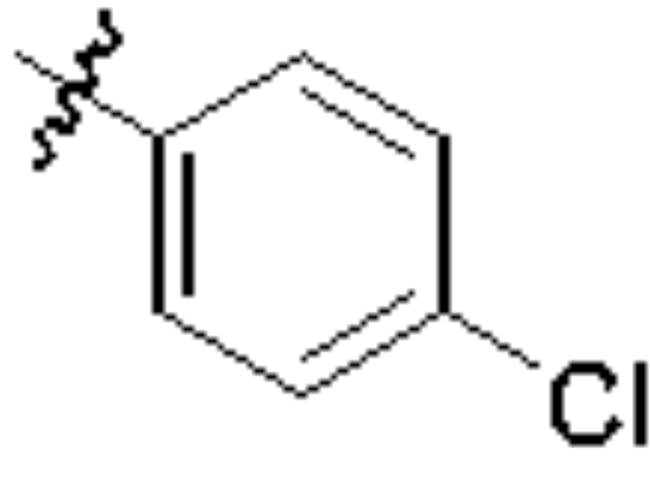	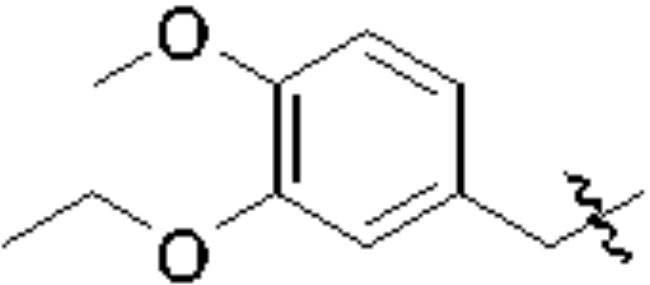	67	F389-0740	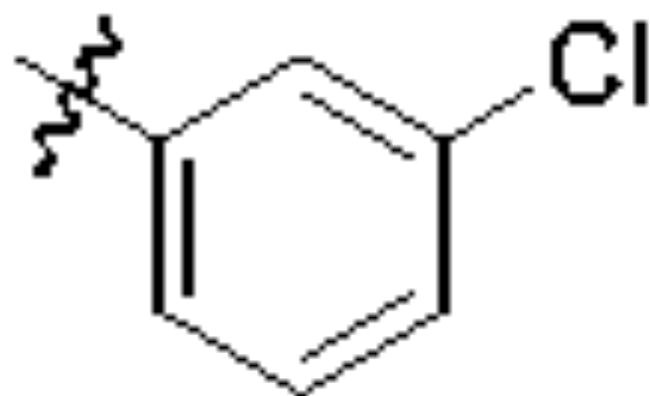	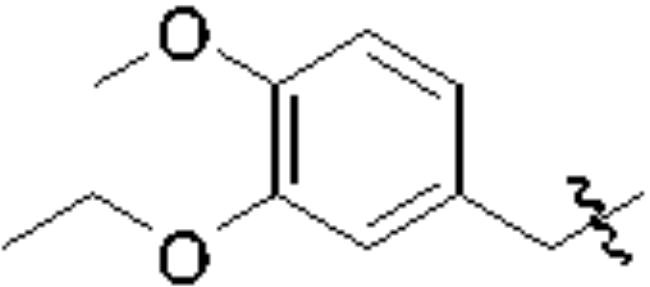	36
F389-0746	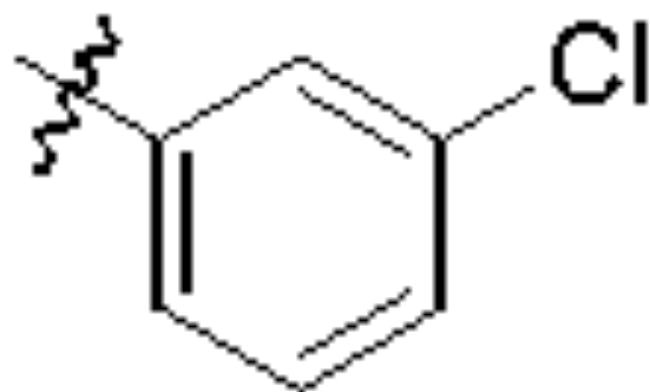	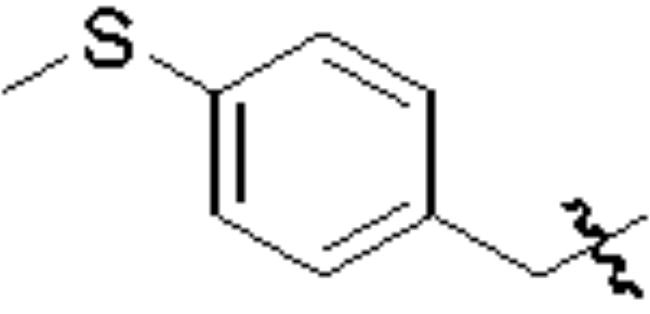	87	F389-0750	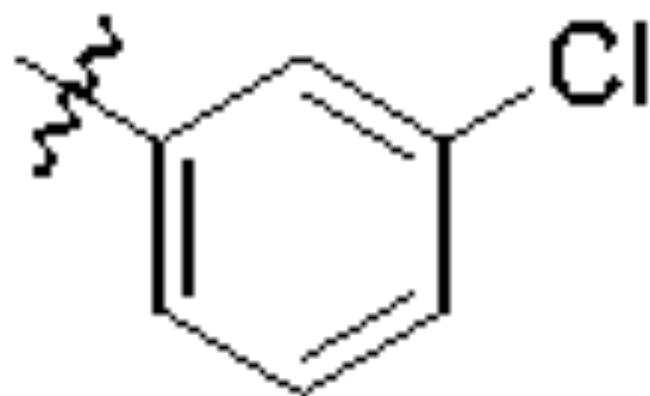	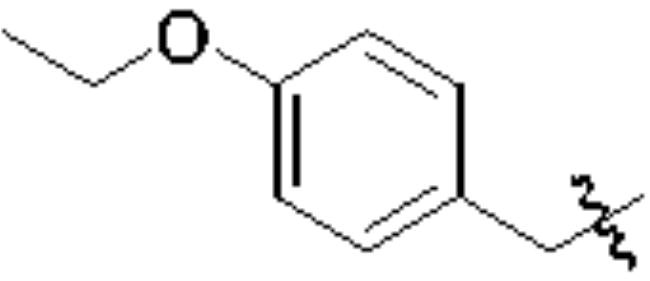	67	F389-0016	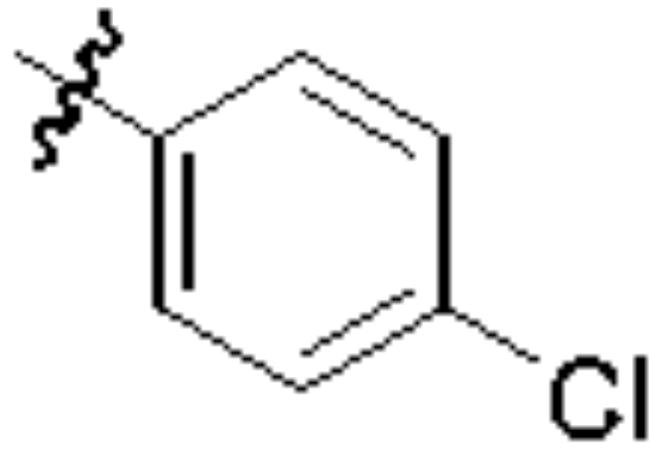	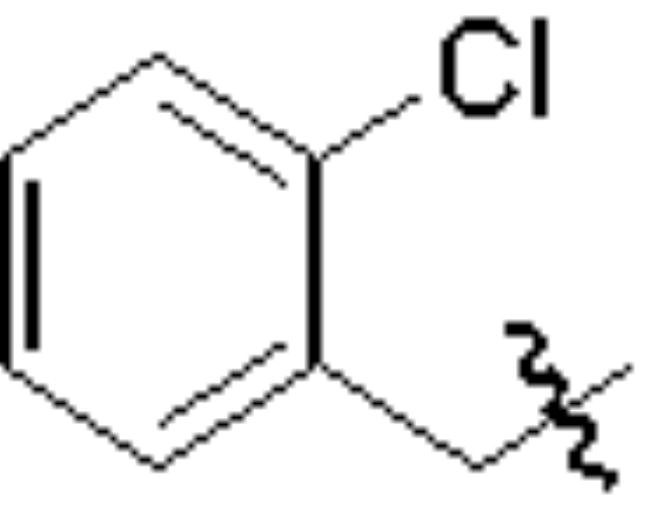	34
F389-0929	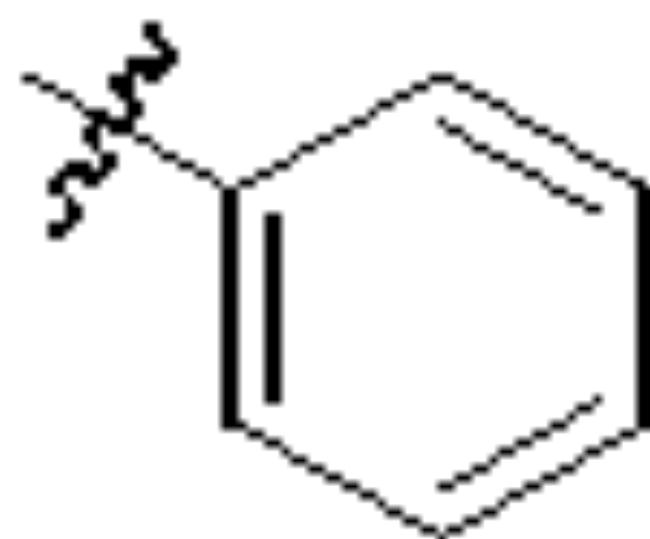	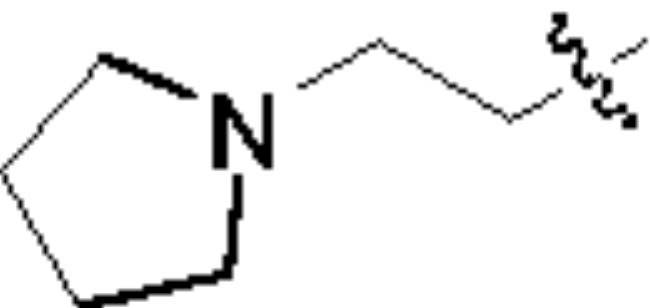	83	F389-0158	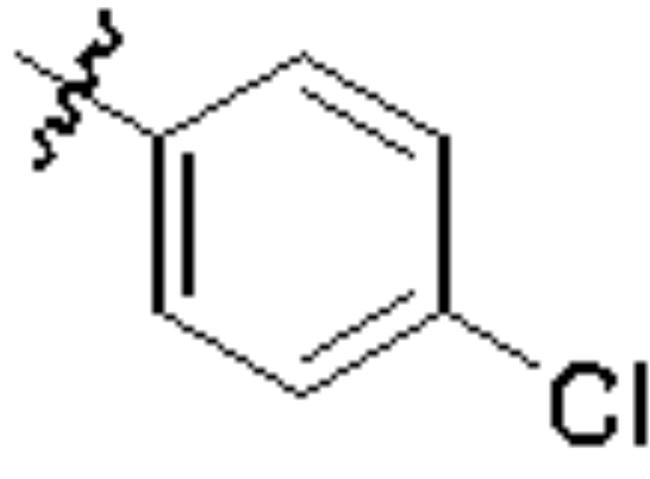	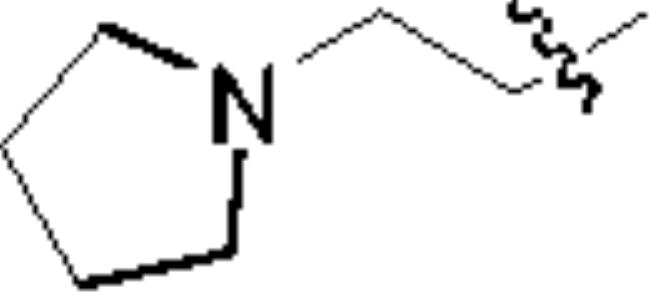	65	F389-0577	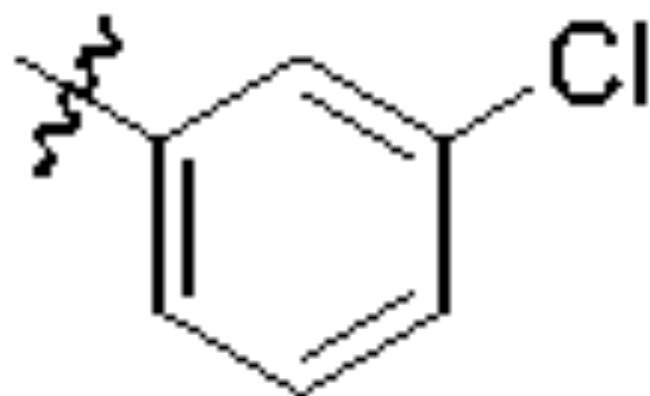	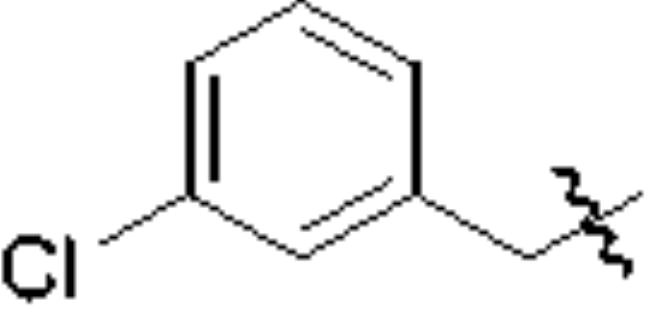	27
F389-0745	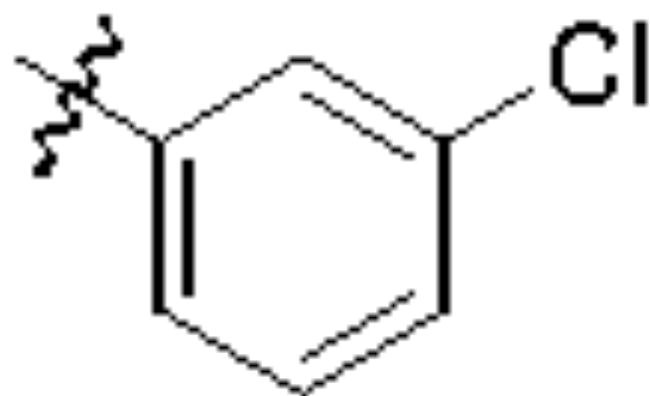	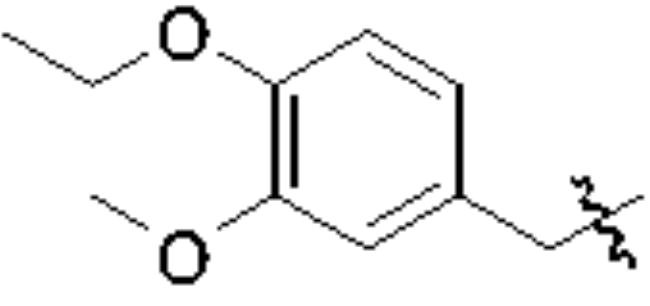	77	F389-0759	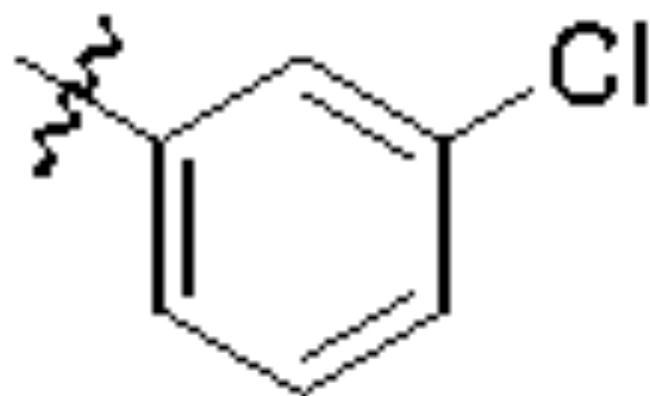	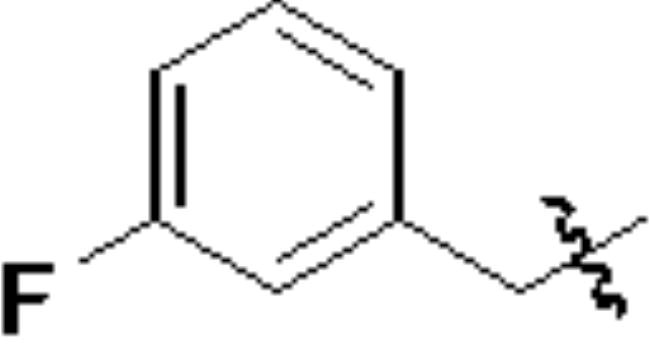	61	F389-0769	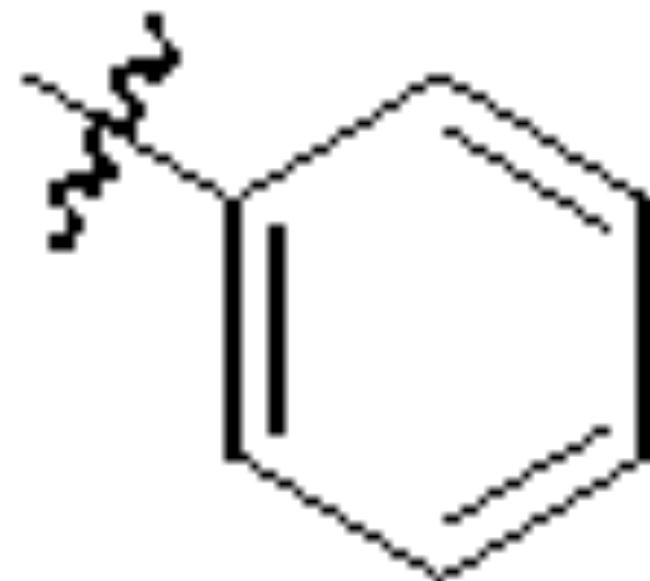	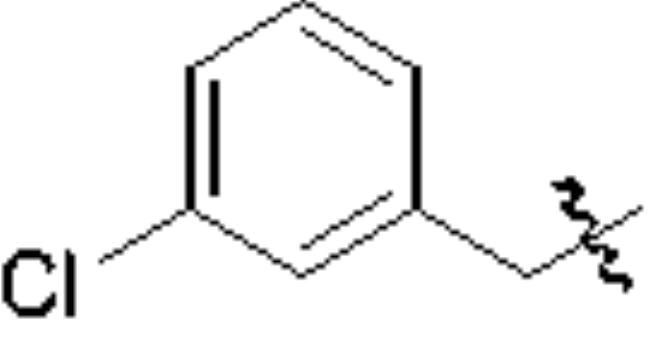	27
F389-0183	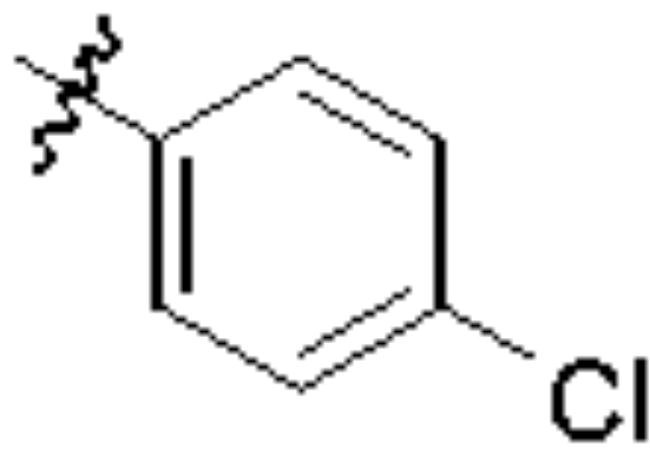	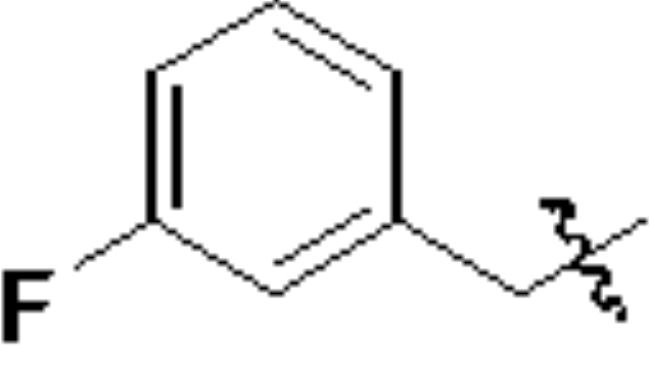	76	F389-0592	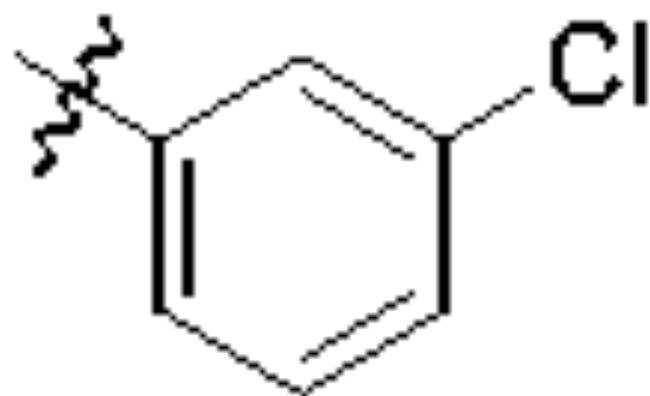	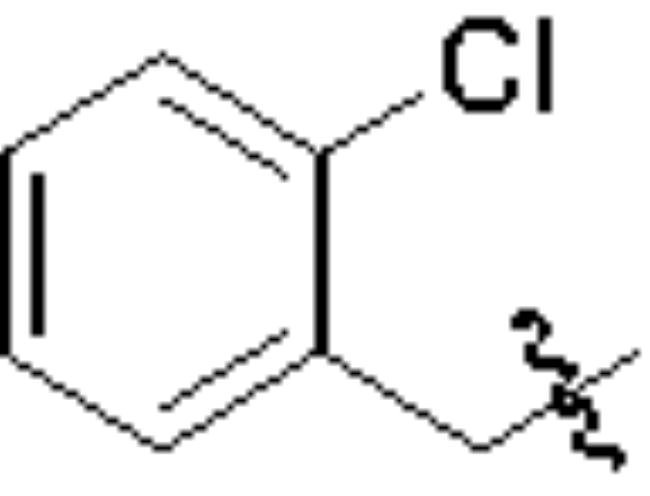	57	F389-0001	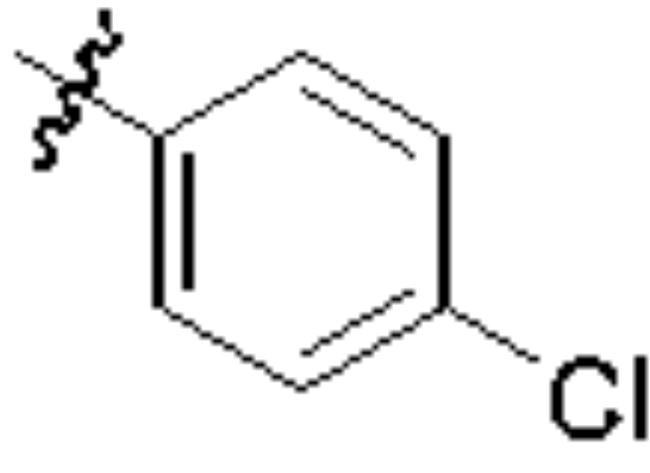	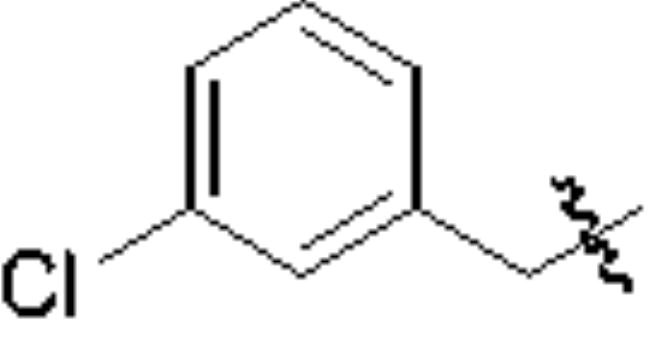	22
F389-0161	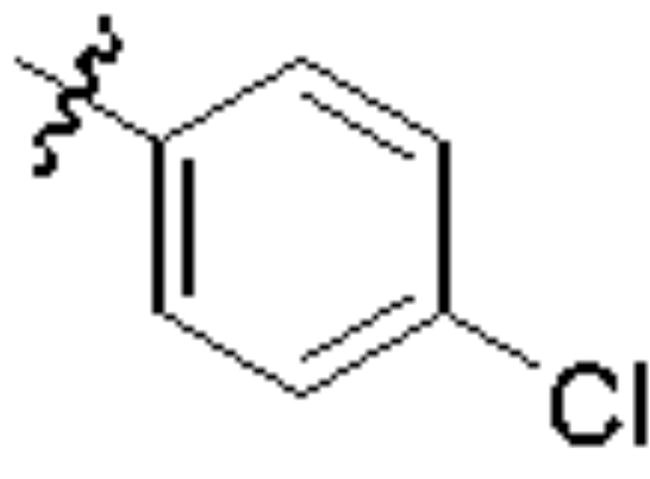	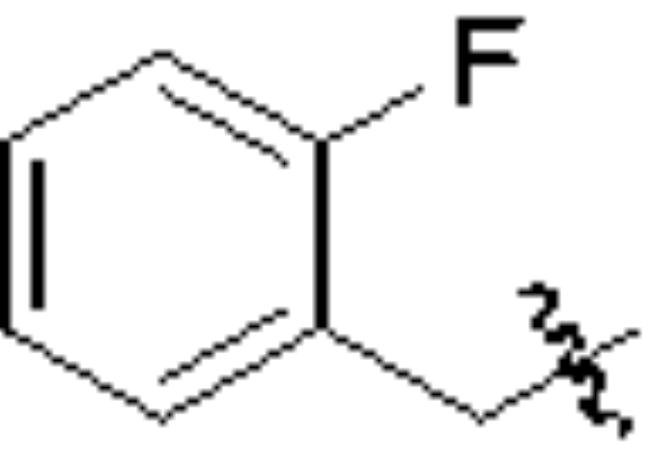	72	F389-0961	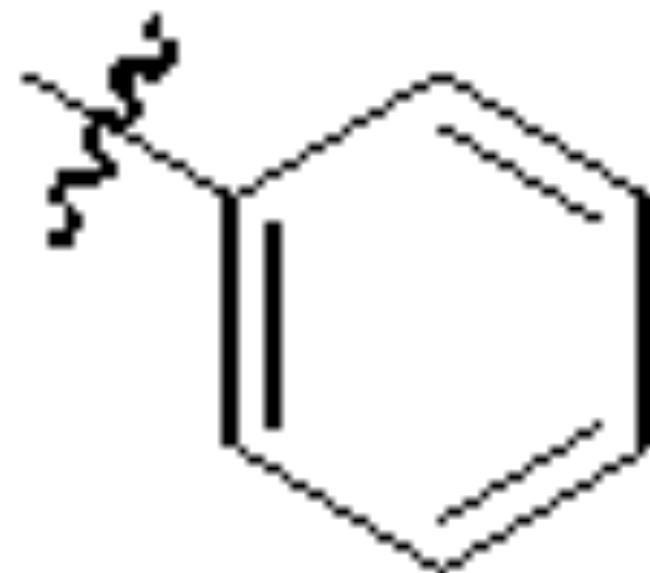	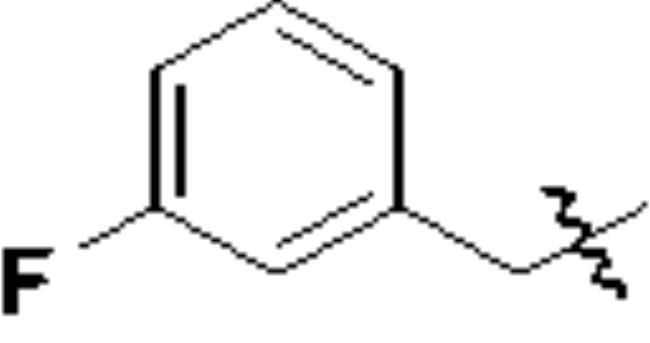	51	F389-0011	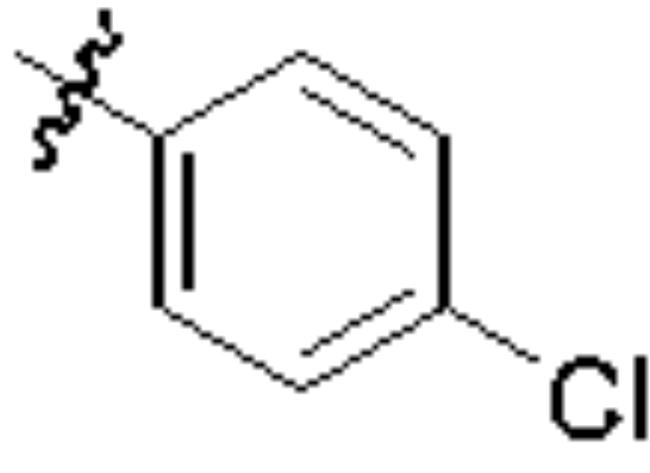	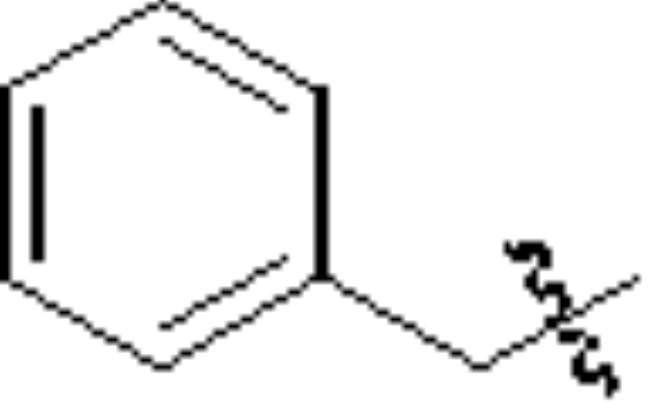	19
F389-0548	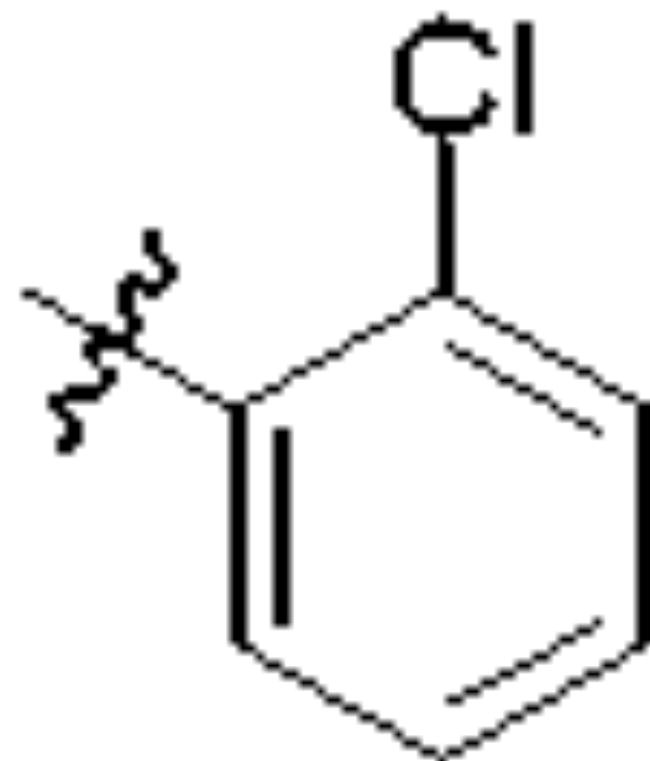	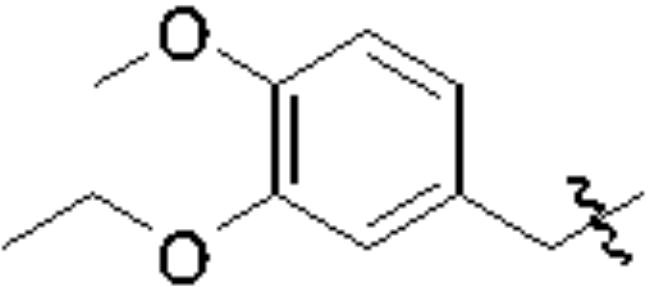	71	F389-0180	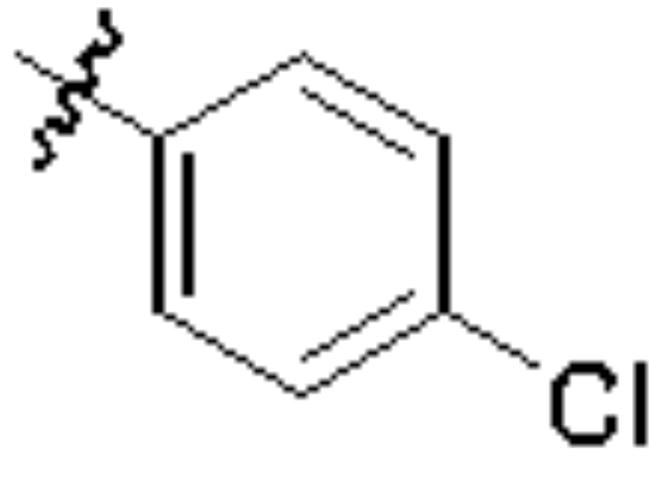	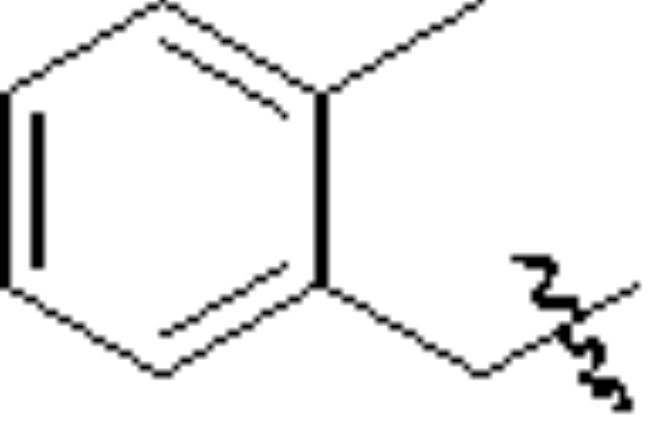	46	F389-0063	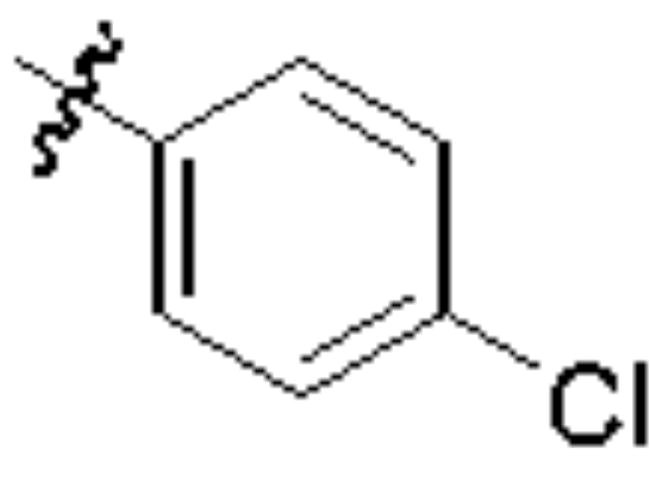	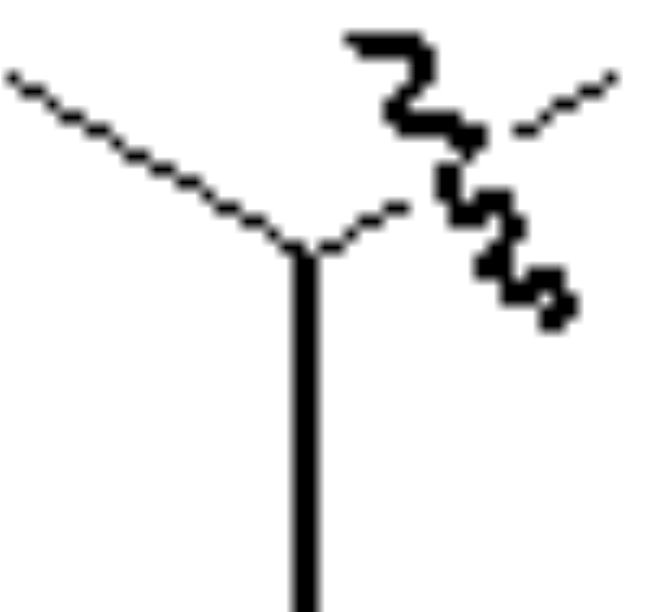	18
F389-0093	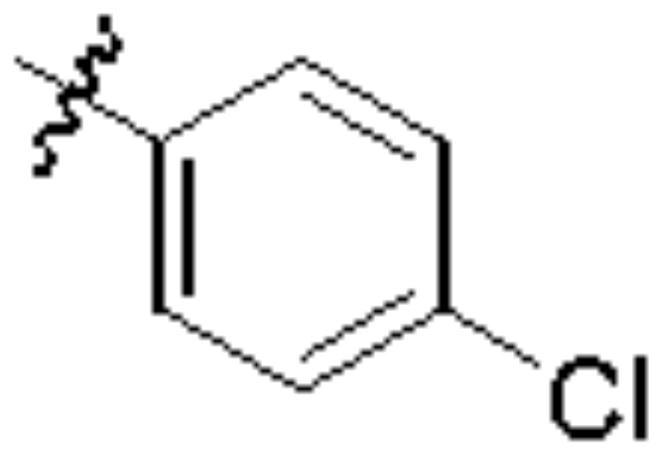	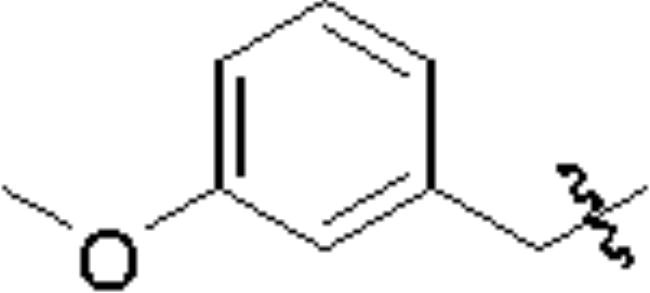	70	F389-0162	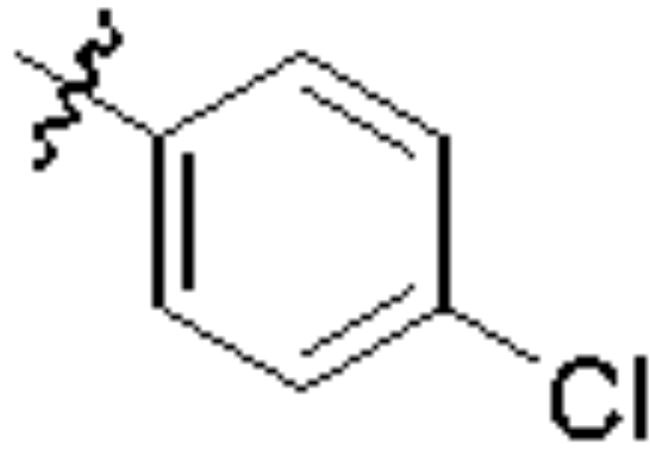	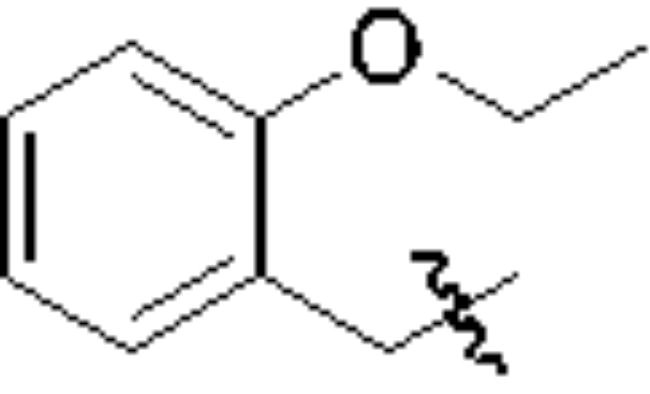	44	F389-0948	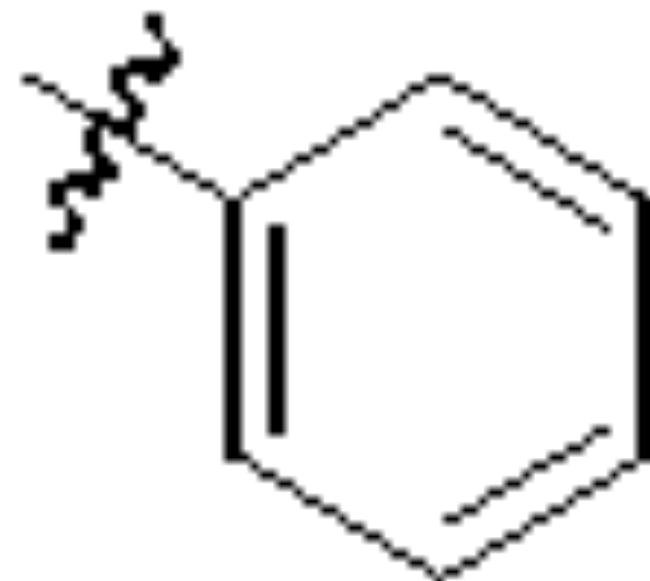	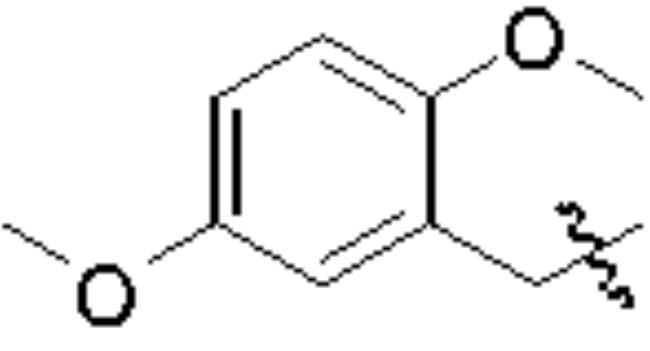	16
								F389-0784	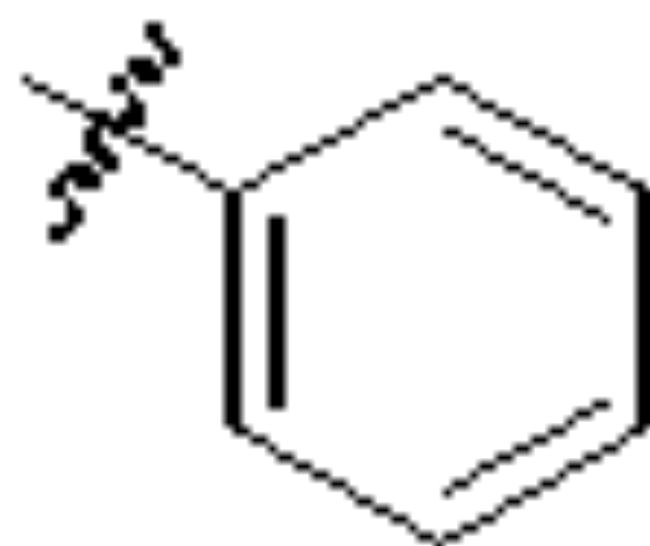	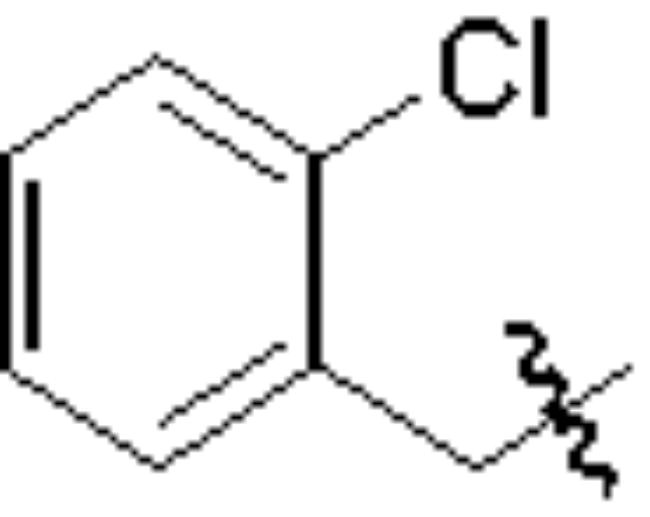	10

Substituents at the R_1_ position also contributed to potency by forming additional hydrophobic contacts in the interior binding pocket. F389-0736 contains a meta-chloride at R_1_, enabling hydrophobic contacts with both K54 and M105. Conversely, F389-0929 with an unsubstituted phenyl ring at R_1_ shows a fourfold higher IC_50_ value than F389-0736. A similar trend was observed when comparing F389-0745 with F389-0944, further confirming that R_1_ chlorination enhances interactions in the interior pocket. Collectively, the SAR analysis demonstrates that R_2_ nitrogen-containing heterocycles enhance potency through salt bridges with D115, while R_1_ chlorobenzene substitution improves activity via hydrophobic contacts with K54 and M105.

The interaction profile of F389-0736 was also compared to molecular docking poses of known MAP4K4 inhibitors (Supplementary Figure 2A). Hydrogen bonds to the kinase hinge remain common for these inhibitors. Similarly, the inhibitors occupy a hydrophobic interior region, which is favourable for the aromatic rings found on the structures (Supplementary Figure 2B). Interactions with the solvent region were found to be important for the F389-0736 analogs. The docking poses of the known MAP4K4 inhibitors also produce interactions with the exterior regions as revealed with residues G32 and D115. These insights provide a foundation for future structure-based optimisation.

### Structural diversity of MAP4K4 inhibitors

A similarity matrix was constructed to assess the structural novelty of F389-0736. The analysis included 30 compounds comprising F389-0736, F389-0746, and 28 structurally diverse known MAP4K4 inhibitors. Pairwise Tanimoto scores were generated for all compound combinations and are provided in Supplementary Figure 3. As expected, F389-0736 and F389-0746 shared the highest mutual similarity as analogs, with a Tanimoto score of 0.630. When compared against known MAP4K4 inhibitors, neither compound demonstrated significant structural overlap with existing inhibitors. The highest-scoring external match was CHEMBL2006765, yielding Tanimoto scores of 0.204 and 0.168 against F389-0736 and F389-0746, respectively. These results demonstrate that the benzoxazine-based series is structurally distinct from previously reported MAP4K4 inhibitors, supporting its novelty within the current MAP4K4 inhibitor landscape.

### Selectivity profile of compound F389-0736

We further evaluated the selectivity of compound F389-0736 by kinase profiling. A panel of kinases representing diverse families across the kinome was selected for testing. At 300 nM, MAP4K4 showed the greatest inhibition, with 17% residual activity, whereas the majority of the other tested kinases retained greater than 50% residual activity (Figure 5). Despite the structural similarity among STE20 family kinase domains, F389-0736 showed negligible inhibition of MAP4K1, PAK4, and STK24, which retained 100%, 88%, and 100% residual activity, respectively. Among kinases positioned near MAP4K4 in the kinome tree, MAP3K9, MYO3A, and MYO3B retained 95%, 100%, and 91% residual activity, respectively, while MINK1 retained 35% residual activity under the same conditions (Figure 5(A)). Concentration response analysis further showed IC_50_ values of 44.0 nM for MAP4K4 and 172.2 nM for MINK1, while the IC_50_ values for MYO3A and MYO3B were both greater than 10 μM (Supplementary Table 1). These findings indicate that F389-0736 is a selective MAP4K4 inhibitor with limited off-target kinase inhibition across the tested panel, making it suitable for mechanistic studies and therapeutic development.

**Figure 5.**
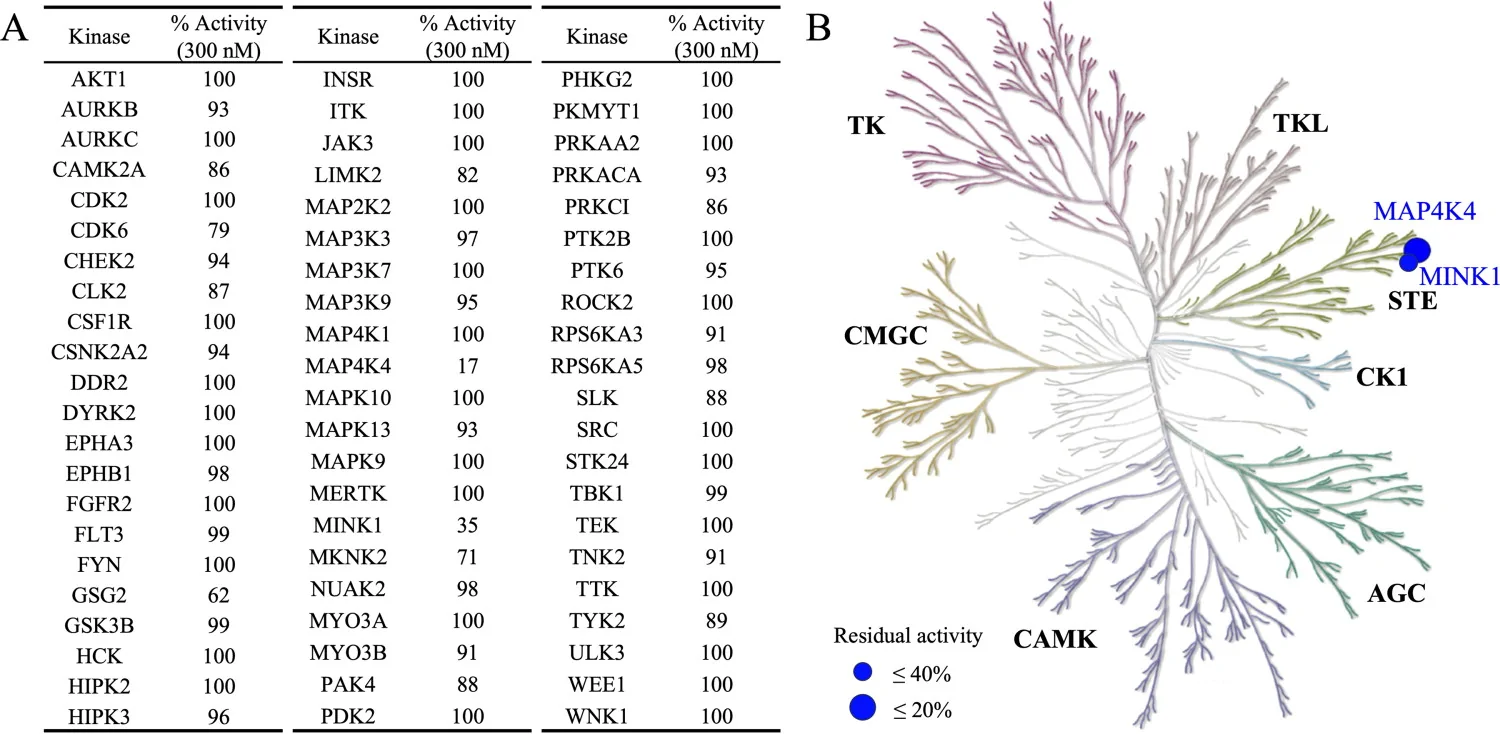
Selectivity profile of compound F389-0736. (A) The residual activity of F389-0736 on a panel of kinases. (B) A kinome tree comprising seven kinase families showing the inhibitory profile of F389-0736. F389-0736 was tested at 300 nM, and MAP4K4 is indicated by a blue dot.

### F389-0736 engages MAP4K4 and suppresses downstream signalling in pancreatic cancer cells

To determine whether F389-0736 engages MAP4K4 in pancreatic cancer cells, CETSA was performed in Mia PaCa-2 cells treated with F389-0736. Compared with the vehicle-treated control, F389-0736 treatment resulted in greater retention of soluble MAP4K4 at higher temperatures, indicating increased thermal stability of MAP4K4. This compound induced thermal stabilisation supports intracellular engagement of F389-0736 with MAP4K4 (Supplementary Figure 4).

We next examined whether F389-0736 modulates MAP4K4-associated signalling in pancreatic cancer cells. Mia PaCa-2 and Panc-1 cells were treated with increasing concentrations of F389-0736 for 4 h, followed by western blot analysis. F389-0736 reduced the phosphorylation levels of TAK1, JNK, and p38 in both cell lines, whereas total JNK and total p38 levels remained relatively unchanged, suggesting that the compound primarily affected the activation status of these signalling proteins rather than their overall protein abundance (Figure 6(A)). Quantitative analysis further showed a concentration-dependent decrease in p-TAK1, p-JNK, and p-p38 levels, with statistically significant inhibition observed at higher concentrations, particularly at 10 μM (Figure 6(B)). These effects were detected in both Mia PaCa-2 and Panc-1 cells, indicating that F389-0736 consistently suppresses TAK1/JNK/p38 pathway activation across pancreatic cancer cell models. GNE-495, included as a reference MAP4K4 inhibitor, similarly reduced the phosphorylation levels of TAK1, JNK, and p38 in both cell lines (Supplementary Figure 5). Together, the CETSA and downstream signalling results support intracellular engagement of MAP4K4 by F389-0736 and the associated suppression of MAP4K4-dependent signalling in pancreatic cancer cells.

**Figure 6.**
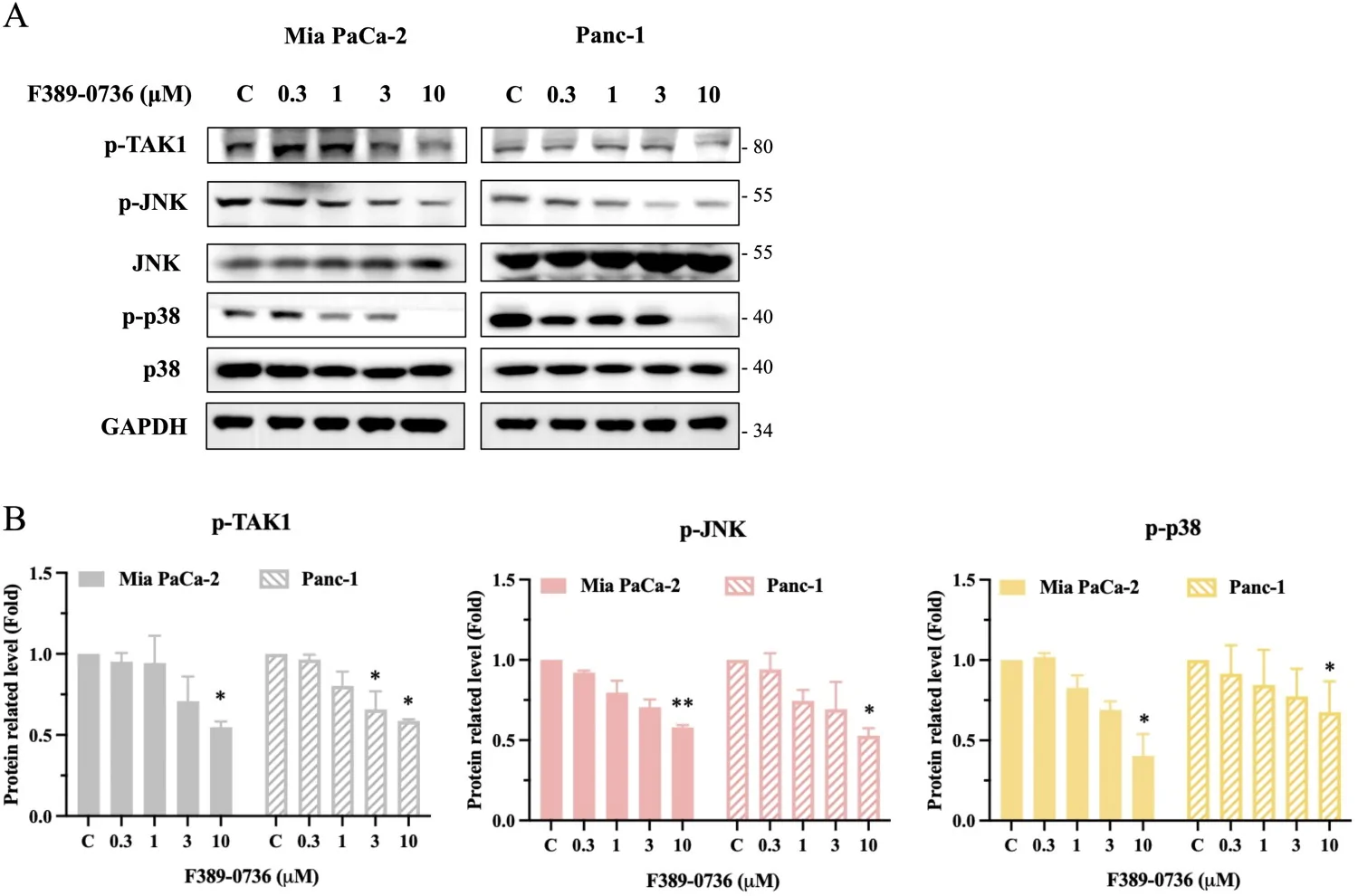
MAP4K4 signalling suppression by F389-0736 in pancreatic cancer cells. Mia PaCa-2 and Panc-1 cells were treated with F389-0736 at 0.3, 1, 3, and 10 μM for 4 h. (A) The phosphorylation levels of TAK1, JNK, and p38 were examined by western blotting. Total JNK, total p38, and GAPDH were used to assess the corresponding phosphorylation levels, and GAPDH served as the loading control. (B) Quantification of p-TAK1, p-JNK, and p-p38 protein levels was performed using ImageJ and normalised to the untreated control group. Data are presented as the mean ± SD from three independent biological experiments (*n* = 3). *p < 0.05, **p < 0.01 compared to the control (C, untreated) group.

### Anti-cancer activity of F389-0736 in pancreatic cancer cells

To evaluate the anti-cancer potential of F389-0736 in pancreatic cancer cells, Mia PaCa-2 and Panc-1 cells were treated with increasing concentrations of F389-0736, followed by assessment of cell viability, proliferation, and clonogenic growth. MTT assay showed that F389-0736 reduced cell viability in both Mia PaCa-2 and Panc-1 cells after 72 h of treatment, with IC_50_ values of 4.41 ± 0.58 μM and 4.65 ± 0.89 μM, respectively (Figure 7(A) and Table 3). Consistently, SRB staining further demonstrated that F389-0736 suppressed cell proliferation in a concentration-dependent manner, with GI_50_ values of 4.03 ± 0.51 μM in Mia PaCa-2 cells and 5.12 ± 1.8 μM in Panc-1 cells (Figure 7(B) and Table 3). For pharmacological comparison, GNE-495 and gemcitabine were also evaluated in parallel, and their cellular activities are summarised in Table 3.

**Figure 7.**
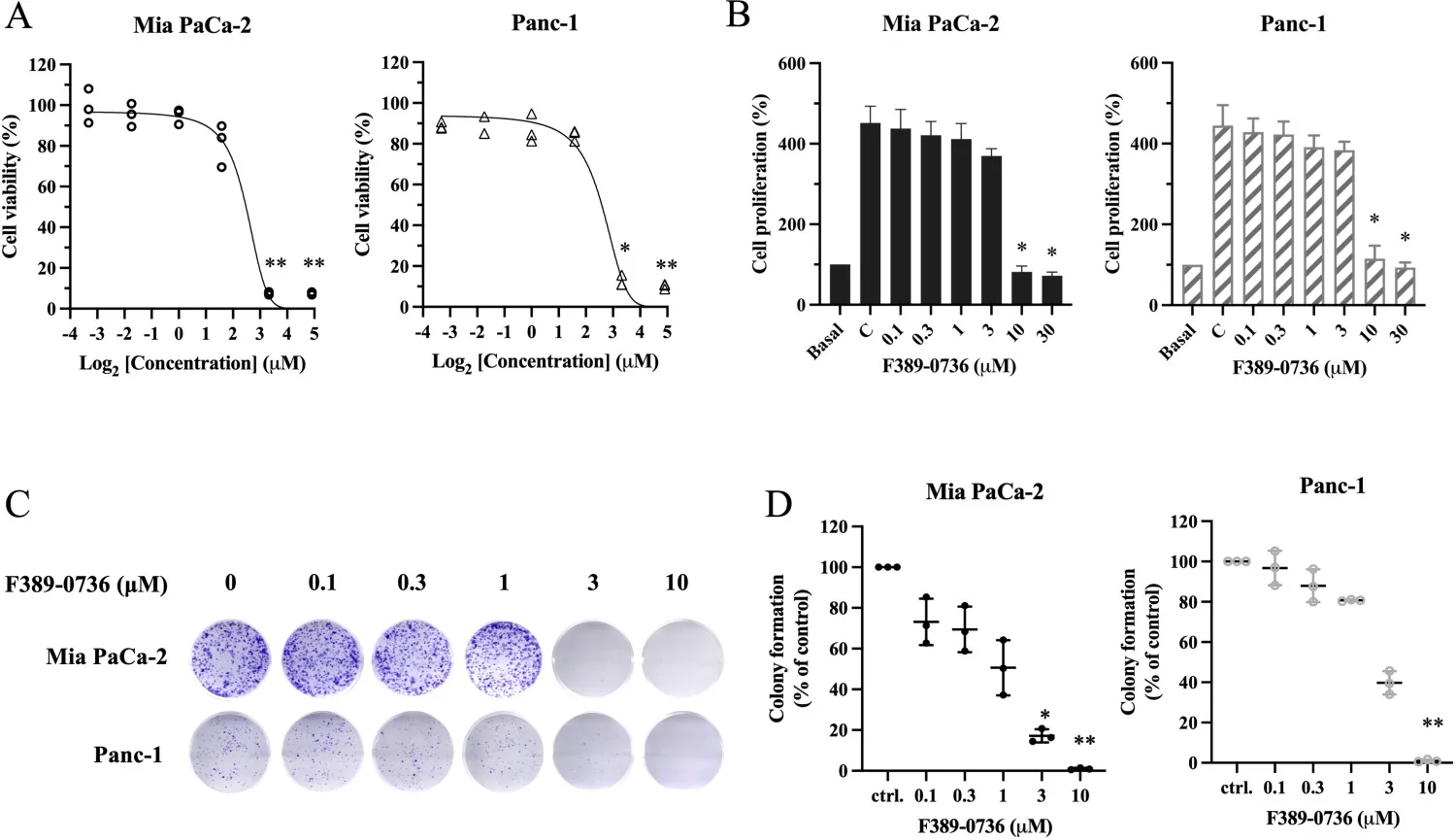
The anti-cancer effect of F389-0736 in pancreatic cancer cells. Mia PaCa-2 and Panc-1 cells were treated with F389-0736 at 0.1, 0.3, 1, 3, 10, and 30 μM for 72 h. (A) Cell viability was determined using the MTT assay. (B) Cell proliferation was evaluated by SRB staining. (C) Long-term clonogenic growth was assessed by colony formation assay. Cells were treated with F389-0736 at 0.1, 0.3, 1, 3, and 10 μM for two weeks, followed by crystal violet staining. (D) Quantification of colony formation. Colonies containing more than 50 cells were counted and normalised to the untreated control group. Data are presented as the mean ± SD from three independent biological experiments (*n* = 3). *p < 0.05, **p < 0.01 compared to the control (C, untreated) group.

**Table 3.** Cellular activity in pancreatic cancer cells.

Compound	Mia PaCa-2		Panc-1	
	**IC_50_ (**μ**M)**	**GI_50_ (**μ**M)**	**IC_50_ (**μ**M)**	**GI_50_ (**μ**M)**
F389-0736	4.41 ± 0.58	4.03 ± 0.51	4.65 ± 0.89	5.12 ± 1.80
GNE-495	> 10	> 10	> 10	> 10
Gemcitabine	3.32 ± 0.05	1.47 ± 0.08	6.37 ± 0.13	1.14 ± 0.08

To further determine whether F389-0736 affects long-term tumour cell growth, colony formation assays were performed. F389-0736 markedly reduced colony formation in Mia PaCa-2 cells, with near-complete inhibition observed at 10 μM. Panc-1 cells also showed a reduction in clonogenic growth, although the inhibitory effect appeared less pronounced at lower concentrations compared with Mia PaCa-2 cells (Figure 7(C)). Quantitative analysis confirmed that F389-0736 significantly decreased colony formation in both pancreatic cancer cell lines at higher concentrations (Figure 7(D)). Gemcitabine was additionally included as a positive control and showed the expected inhibition of colony formation in both cell lines (Supplementary Figure 6). Collectively, these findings indicate that F389-0736 exhibits anti-cancer activity by suppressing pancreatic cancer cell viability, proliferation, and long-term clonogenic capacity.

### F389-0736 induces Sub-G1 accumulation and apoptotic signalling

Given the marked suppression of cell viability and clonogenic growth, the effect of F389-0736 on cell cycle distribution and apoptosis was subsequently investigated. After 72 h of treatment, F389-0736 altered cell cycle distribution in both Mia PaCa-2 and Panc-1 cells, with a marked increase in the sub-G1 population at 10 μM (Figure 8(A)). Quantitative analysis further confirmed that 10 μM F389-0736 significantly increased the percentage of sub-G1 cells in both pancreatic cancer cell lines, indicating the induction of apoptotic cell death (Figure 8(B)). Consistently, western blot analysis showed that F389-0736 decreased the expression of the anti-apoptotic protein Bcl-2 and increased the levels of cleaved PARP and cleaved caspase-3, accompanied by reduced caspase-9 and caspase-3 levels, particularly at 10 μM (Figure 8(C)). Densitometric quantification further supported these changes, showing significant downregulation of Bcl-2 and caspase-9 and upregulation of cleaved PARP and cleaved caspase-3 after F389-0736 treatment (Supplementary Figure 7). Gemcitabine was additionally included as a positive control and induced corresponding apoptosis associated changes in both pancreatic cancer cell lines (Supplementary Figure 8). Collectively, these findings indicate that F389-0736 promotes sub-G1 accumulation and activates mitochondria mediated apoptotic signalling in pancreatic cancer cells.

**Figure 8.**
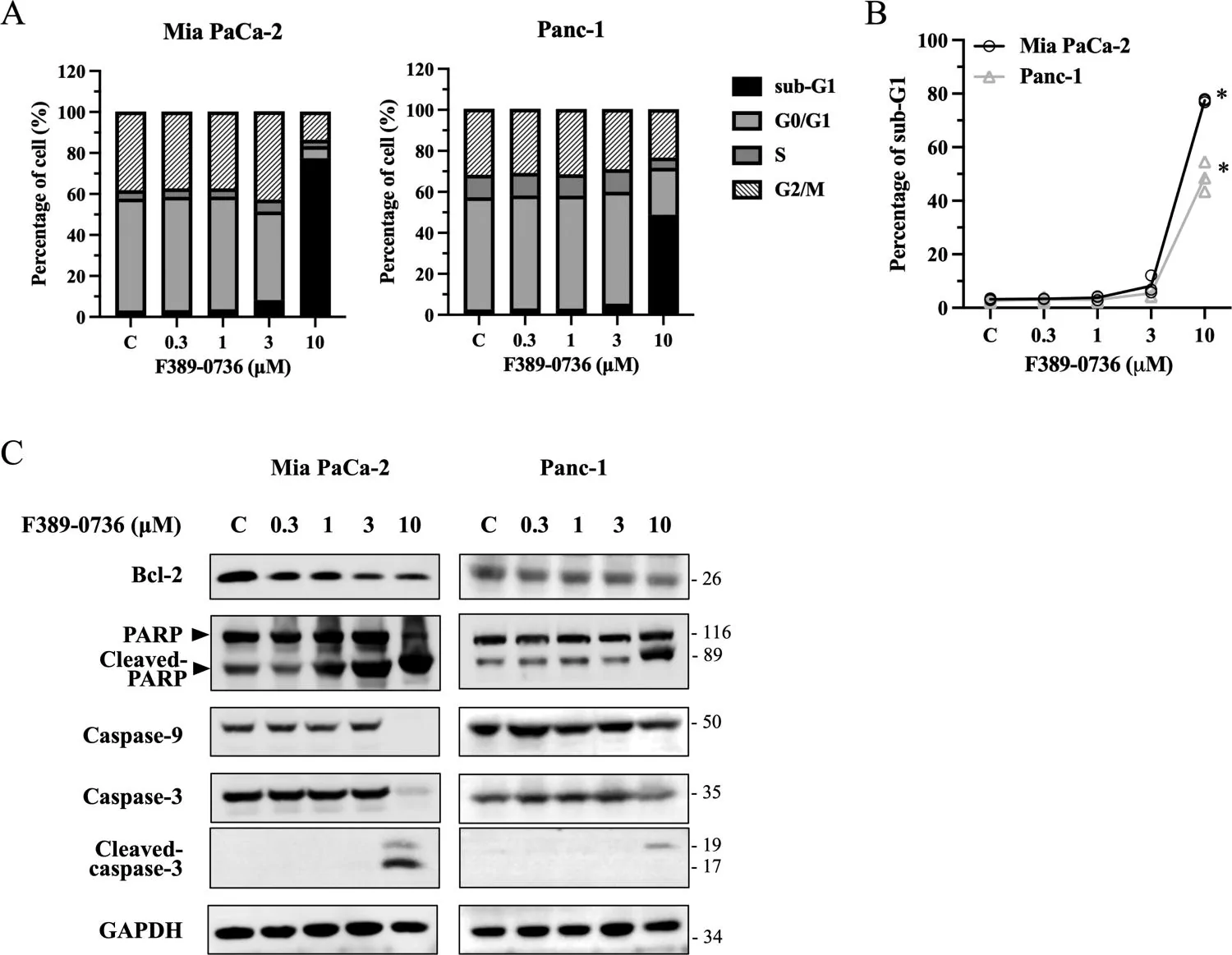
Induction of sub-G1 accumulation and apoptosis by F389-0736 in pancreatic cancer cells. Mia PaCa-2 and Panc-1 cells were treated with F389-0736 at 0.3, 1, 3, and 10 μM for 72 h. (A) Cell cycle distribution was analysed by flow cytometry. (B) Quantification of the sub-G1 population in Mia PaCa-2 and Panc-1 cells. (C) The expression of apoptosis-related proteins, including Bcl-2, PARP, caspase-9, caspase-3, and cleaved caspase-3, was examined by western blotting. GAPDH was used as a loading control. Data are presented as the mean ± SD from three independent biological experiments (*n* = 3). *p < 0.05 compared with the untreated control (C) group.

### Transcriptomic signatures associated with F389-0736 mediated pancreatic cancer cell suppression

To characterise the molecular consequences of MAP4K4 inhibition, RNA sequencing was performed in Mia PaCa-2 cells treated with F389-0736, followed by differential gene expression and pathway enrichment analyses. Using the criteria of |log_2_FC| > 2 and adjusted *p* < 0.01, a total of 398 differentially expressed genes (DEGs) were identified, indicating substantial transcriptomic reprogramming following MAP4K4 inhibition (Figure 9(A)). The volcano plot revealed a prominent induction of inflammatory and stress response genes, including *TNF, TRAF1, TNFRSF9, NFKB2, IL1A, IL1B, CXCL8, BIRC3, CSF3, and HMOX1*. These genes are associated with TNF/NF-κB signalling, cytokine-mediated responses, and cellular stress adaptation. Because the analysis was performed in cultured Mia PaCa-2 cells, these immune-related signatures likely reflect a tumour-cell-intrinsic inflammatory stress response rather than immune-cell infiltration. Notably, *HMOX1* induction suggests oxidative stress and redox remodelling, which may further contribute to ferroptosis-associated vulnerability under sustained stress.^27^

**Figure 9.**
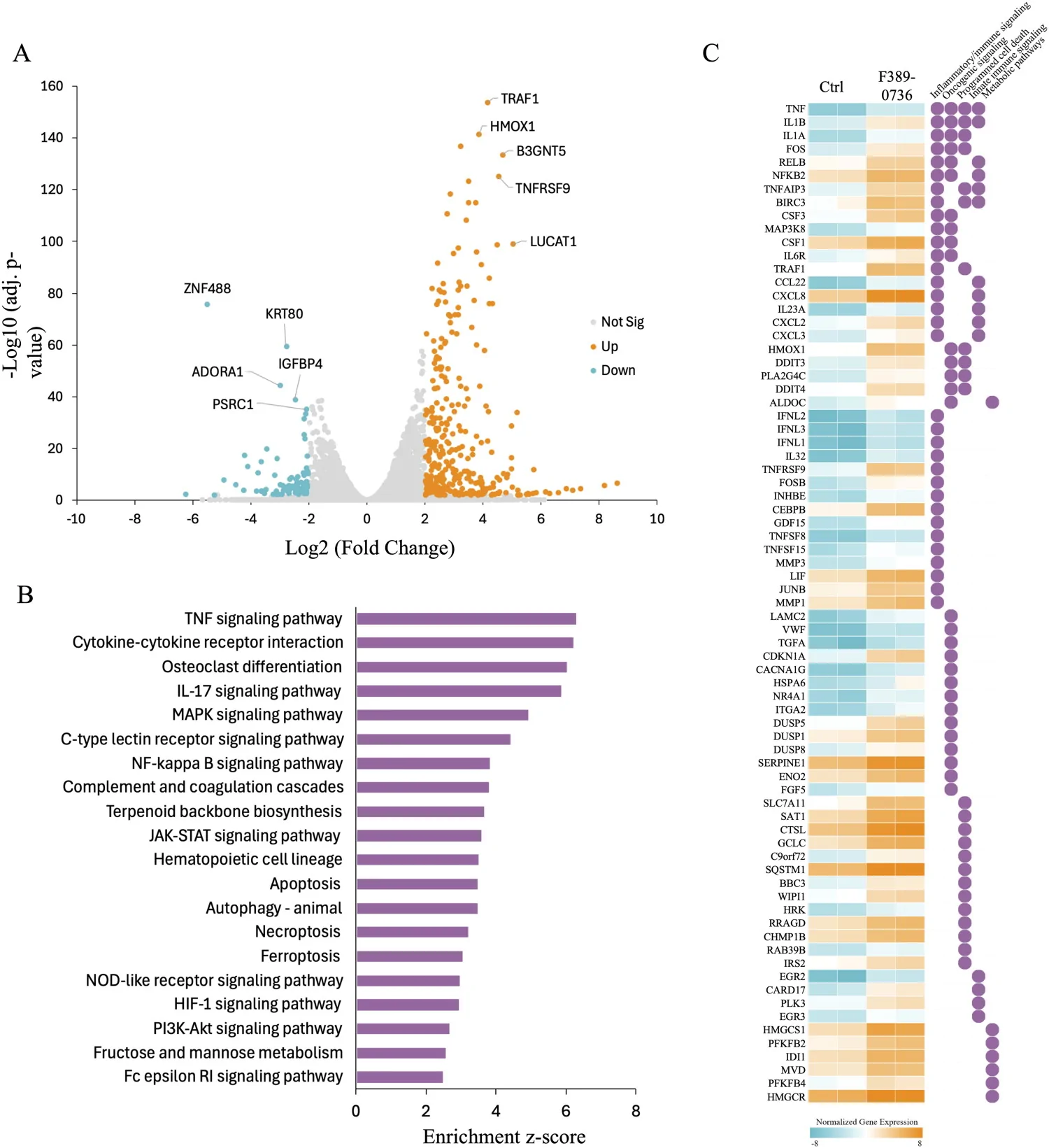
Transcriptomic profiling and pathway enrichment analysis following MAP4K4 inhibition. Mia PaCa-2 cells were treated with F389-0736 for 24 h, followed by RNA sequencing analysis using two biological replicates per group (*n* = 2). (A) Differential gene expression analysis of F389-0736-treated cells compared with control cells. The volcano plot shows DEGs identified using |log_2_ fold change| > 2 and adjusted *p* < 0.01. Upregulated genes are shown in orange, downregulated genes in cyan, and non-significant genes in gray. Representative upregulated stress response genes, including *TRAF1, HMOX1, B3GNT5, TNFRSF9, and LUCAT1*, and downregulated tumour-associated genes, including *ZNF488, KRT80, ADORA1, IGFBP4, and PSRC1*, are labelled. (B) Pathway enrichment analysis. Top enriched KEGG pathways based on DEGs following F389-0736 treatment, ranked by enrichment z-score. Pathways are grouped into five functional categories, including inflammatory/immune signalling (TNF, NF-κB, IL-17, cytokine–cytokine receptor interaction, JAK-STAT), innate immune signalling (NOD-like receptor and C-type lectin receptor), programmed cell death (apoptosis, ferroptosis, necroptosis, autophagy), oncogenic signalling (MAPK, PI3K-Akt, HIF-1), and metabolic pathways (terpenoid backbone biosynthesis, fructose and mannose metabolism). (C) Heatmap and pathway-membership summary of representative DEGs contributing to the enriched functional pathways. The heatmap displays relative expression differences between control and F389-0736-treated cells, while purple circles indicate the pathways associated with each gene.

F389-0736 treatment also induced immediate early response transcription factors, including *FOS, JUNB, EGR1-4, and ATF3*, suggesting activation of stress-responsive transcriptional programs downstream of MAPK/JNK pathway perturbation.^28^ This transcriptional response is consistent with the observed suppression of TAK1, JNK, and p38 phosphorylation, indicating that MAP4K4 inhibition disrupts stress kinase signalling while triggering compensatory or downstream stress-adaptive gene expression. Conversely, several tumour-associated genes were significantly downregulated, including *ZNF488, KRT80, ADORA1, IGFBP4, and PSRC1* (Figure 9(A)). Suppression of these genes may contribute to the anti-proliferative effects of F389-0736. For example, *ZNF488* has been linked to tumour progression and ferroptosis suppression, whereas *KRT80* is associated with epithelial–mesenchymal transition and invasive phenotypes in multiple cancers.^29^^,^^30^ These changes support the notion that MAP4K4 inhibition suppresses pro-tumorigenic transcriptional programs while inducing stress-associated responses.

KEGG pathway enrichment analysis further showed that the DEGs were organised into several functional modules (Figure 9(B)). The most enriched pathways included TNF signalling, cytokine-cytokine receptor interaction, IL-17 signalling, NF-κB signalling, JAK-STAT signalling, NOD-like receptor signalling, and C-type lectin receptor signalling. These results indicate coordinated activation of inflammatory, cytokine-response, and innate immune-like stress pathways within pancreatic cancer cells. Pathways such as osteoclast differentiation and haematopoietic cell lineage were also enriched, likely due to shared cytokine and NF-κB-associated genes rather than lineage-specific differentiation.

Importantly, multiple programmed cell death-associated pathways, including apoptosis, ferroptosis, necroptosis, and autophagy, were concurrently enriched (Figure 9(B)). Among these, apoptosis is supported by the functional data showing sub-G1 accumulation, Bcl-2 downregulation, PARP cleavage, and caspase-3 activation after F389-0736 treatment (Figure 8(C)). The enrichment of ferroptosis, necroptosis, and autophagy suggests additional stress-associated cytotoxic programs that may contribute to reduced pancreatic cancer cell survival, although further functional validation would be required. In addition, MAP4K4 inhibition perturbed key oncogenic and adaptive signalling pathways, including MAPK, PI3K-Akt, and HIF-1 signalling, which regulate proliferation, survival, hypoxia adaptation, and metabolic fitness in pancreatic cancer cells. Enrichment of metabolic pathways such as terpenoid backbone biosynthesis and fructose and mannose metabolism further suggests metabolic remodelling in response to F389-0736-induced cellular stress.^31^

The heatmap and functional pathway analysis further integrated these findings at the gene level (Figure 9(C)). Several representative DEGs showed overlapping membership across inflammatory signalling, cytokine response, programmed cell death, oncogenic signalling, and metabolic pathways, suggesting that F389-0736 induces an integrated transcriptional stress network rather than isolated pathway-specific changes. Overall, these transcriptomic results demonstrate that MAP4K4 inhibition by F389-0736 induces tumour-cell-intrinsic inflammatory activation, suppresses tumour-associated gene programs, perturbs survival and metabolic signalling, and enriches multiple programmed cell death-associated pathways, providing mechanistic insight into its anti-tumour activity in pancreatic cancer cells.

## Discussion

In this study, we identified a more potent benzoxazine-based MAP4K4 inhibitor, F389-0736, with an IC_50_ value of 44 nM through screening analogs of a compound library. The potency of F389-0736 differed between the enzymatic assay and the cellular assay. This can be attributed to the “cell drop off” phenomenon.^32–34^ In short, a higher dosage would be required to produce the desired inhibition in the cell environment. Nevertheless, F389-0736 produced favourable IC_50_ values of 4.41 µM and 4.65 µM in Mia PaCa-2 and Panc-1 cell lines, respectively. This analogue demonstrated high kinase selectivity and potent anti-cancer activity. Importantly, SAR analysis revealed key structural determinants of MAP4K4 inhibitory potency, providing a clear framework for rational inhibitor design. One critical determinant was that nitrogen-containing heterocycles at Group 1 forming multiple hydrogen bonds with D115 conferred higher potency than substituents interacting with R310. This observation explains the superior activity of compounds such as F389-0736 and F389-0743. These findings collectively define the optimal pharmacophore for MAP4K4 inhibition, comprising a nitrogen-containing heterocycle at Group 1, an amide linker at Group 2, a benzoxazine core at Group 3, and a chlorobenzene substituent at Group 4.

To compare the binding profile of F389-0736 with known MAP4K4 inhibitors, we analysed its protein-ligand interactions with 15 known MAP4K4 inhibitors, including five representative compounds: Famlasertib,^35^ DMX-5804,^36^ GNE-495,^17^ PF-06745013,^18^ and CHEMBL3326802.^19^ Over 85% of inhibitors, including F389-0736, engaged conserved residues in the hinge and interior regions. V31, V39, and A52 on the β-sheets forming the ceiling of the binding pocket and L160 and V170 on the floor sandwiched the heterocyclic scaffolds through multiple hydrophobic interactions. The hinge residues F107 and C108 anchored inhibitors through hydrogen bonds. These conserved interactions represent essential features of MAP4K4 inhibitors. Beyond these common interactions, F389-0736 exhibits two distinctive features associated with enhanced potency. First, similar to Famlasertib, F389-0736 forms hydrogen bonds with D115 through its nitrogen-containing heterocycle extending into the solvent-exposed region (Supplementary Figure 2B). This interaction aligns with our SAR analysis showing that D115 engagement confers superior potency. Second, F389-0736 forms hydrophobic interactions with K54, an interaction observed in fewer than 50% of inhibitors but present in nearly all lead compounds. Together, these observations suggest that engagement of D115 and K54 may be associated with high inhibitory potency in MAP4K4 inhibitors.

RNA-seq analysis further expanded the mechanistic understanding of F389-0736-mediated MAP4K4 inhibition. F389-0736 induced broad transcriptional reprogramming characterised by activation of tumour-cell-intrinsic inflammatory and stress response programs, particularly TNF/NF-κB-associated gene networks. This response may reflect a stress-adaptive transcriptional state triggered by disruption of MAP4K4-dependent survival signalling rather than immune-cell-derived inflammation. Consistent with the observed induction of apoptosis, pathway enrichment analysis also revealed multiple programmed cell death-associated signatures, including apoptosis, ferroptosis, necroptosis, and autophagy. Among these, apoptosis was supported by functional validation, including sub-G1 accumulation, Bcl-2 downregulation, PARP cleavage, and caspase-3 activation, whereas ferroptosis, necroptosis, and autophagy may represent additional stress-associated vulnerabilities induced by MAP4K4 inhibition.^27^ In parallel, perturbation of MAPK, PI3K-Akt, HIF-1, and metabolic pathways suggests that MAP4K4 regulates a broader signalling network involved in tumour cell survival, stress tolerance, and metabolic adaptation.^28^^,^^31^ Together, these findings support a model in which F389-0736 compromises pancreatic cancer cell fitness by suppressing MAP4K4-dependent kinase signalling while inducing inflammatory stress responses and cell death-associated transcriptional programs.

To further assess the drug-like properties of F389-0736, *in silico* ADMET prediction was performed using the Deep-PK platform (Supplementary Table 2).^37^ F389-0736 showed physicochemical properties generally consistent with a drug-like profile and favourable predictions for absorption, including a high probability of human intestinal absorption and oral bioavailability. The predicted metabolic profile also indicated relatively low probabilities of inhibition across the major CYP isoforms. However, the high predicted plasma protein binding and an elimination half-life of less than 3 h indicate pharmacokinetic properties that warrant further experimental evaluation and potential areas for optimisation. In addition, F389-0736 showed low predicted probabilities of Ames mutagenicity, carcinogenicity, and drug-induced liver injury, providing preliminary support for its safety profile. Nevertheless, these computational predictions are not a substitute for experimental pharmacokinetic and toxicity assessments, and the absence of *in vivo* anti-tumour efficacy, pharmacokinetic, and toxicity studies remains a limitation of the present study. Further studies are therefore required to experimentally characterise the pharmacokinetic and safety profiles of F389-0736 and evaluate its anti-tumour efficacy in pancreatic cancer models.

## Conclusion

This study identifies F389-0736 as a potent and selective benzoxazine-based MAP4K4 inhibitor with anti-cancer activity in pancreatic cancer cells. F389-0736 showed improved MAP4K4 inhibitory potency compared with the previous compound F389-0746, supported by key D115 interactions and hydrophobic contacts within the MAP4K4 binding pocket. CETSA further demonstrated increased thermal stability of MAP4K4 following F389-0736 treatment, supporting intracellular target engagement. Functionally, F389-0736 suppressed TAK1/JNK/p38 signalling, reduced cell viability, proliferation, and clonogenic growth, and induced sub-G1 accumulation with activation of apoptotic markers. Transcriptomic analysis further revealed broad molecular reprogramming involving stress response, programmed cell death, oncogenic signalling disruption, and metabolic adaptation. Together, these findings support F389-0736 as a useful chemical probe for investigating MAP4K4-dependent signalling and as a promising lead scaffold for developing selective MAP4K4 targeted strategies in pancreatic cancer.

## Supplementary Material

GENZ-2026-0341.R1_Editable Tables_Cl.docx
