# Supplementary material for: Optimisation and mechanistic characterization of a selective benzoxazine-based MAP4K4 inhibitor in pancreatic cancer cells
Source: J Enzyme Inhib Med Chem. 2026 Oct 16;41(1):2743434. doi: 10.1080/14756366.2026.2743434 (PMC13678883; doi:10.1080/14756366.2026.2743434)
Supplement: Supplementary file 1 [file IENZ_A_2743434_SM5149.docx]

**Table 1. IC_50_ values of identified analogs.**

| Name | IC_50_ (nM) |
| --- | --- |
| F389-0736   | 44.0 |
| F389-0743   | 86.9 |
| F389-0929   | 184.7 |

**Table 2. Inhibitory activity of F389-0736 analogs.**

|  | | | | | | | | | | | | |
| --- | --- | --- | --- | --- | --- | --- | --- | --- | --- | --- | --- | --- |
| Compound | R_1_ | R_2_ | % Inhibition  (1 μM) | Compound | R_1_ | R_2_ | % Inhibition  (1 μM) | Compound | R_1_ | R_2_ | % Inhibition  (1 μM) |  |
| F389-0736 |  |  | 93 | F389-0559 |  |  | 70 | F389-0944 |  |  | 40 |  |
| F389-0743 |  |  | 88 | F389-0164 |  |  | 67 | F389-0740 |  |  | 36 |  |
| F389-0746 |  |  | 87 | F389-0750 |  |  | 67 | F389-0016 |  |  | 34 |  |
| F389-0929 |  |  | 83 | F389-0158 |  |  | 65 | F389-0577 |  |  | 27 |  |
| F389-0745 |  |  | 77 | F389-0759 |  |  | 61 | F389-0769 |  |  | 27 |  |
| F389-0183 |  |  | 76 | F389-0592 |  |  | 57 | F389-0001 |  |  | 22 |  |
| F389-0161 |  |  | 72 | F389-0961 |  |  | 51 | F389-0011 |  |  | 19 |  |
| F389-0548 |  |  | 71 | F389-0180 |  |  | 46 | F389-0063 |  |  | 18 |  |
| F389-0093 |  |  | 70 | F389-0162 |  |  | 44 | F389-0948 |  |  | 16 |  |
|  |  |  |  |  |  |  |  | F389-0784 |  |  | 10 |  |

**Table 3. Cellular activity in pancreatic cancer cells.**

| **Compound** | **Mia PaCa-2** | | **Panc-1** | |
| --- | --- | --- | --- | --- |
|  | **IC_50_ (**μ**M)** | **GI_50_ (**μ**M)** | **IC_50_ (**μ**M)** | **GI_50_ (**μ**M)** |
| F389-0736 | 4.41 ± 0.58 | 4.03 ± 0.51 | 4.65 ± 0.89 | 5.12 ± 1.80 |
| GNE-495 | > 10 | > 10 | > 10 | > 10 |
| Gemcitabine | 3.32 ± 0.05 | 1.47 ± 0.08 | 6.37 ± 0.13 | 1.14 ± 0.08 |

**Supplementary Table 1. IC_50_ values of F389-0736 against selected kinases.**

| Kinase | IC_50_ (nM) |
| --- | --- |
| MAP4K4 | 44.0 |
| MINK1 | 172.2 |
| MYO3A | >10000 |
| MYO3B | >10000 |

**Supplementary Table 2. Predicted drug-like and ADMET properties of F389-0736.**

| **Physicochemical Property** | | |
| --- | --- | --- |
|  | **Property** | **Value** |
|  | Molecular Weight (MW) | 454.955 |
|  | LogP | 2.59 |
|  | LogS (logmol/L) | -3.29 |
| **ADMET prediction** | | |
|  | **Property** | **Probability / Value** |
| Absorption | Human Intestinal Absorption (HIA) | 0.98 |
|  | Human Oral Bioavailability | 0.70 |
|  | Caco-2 Permeability (logP_app_) | -5.37 |
| Distribution | Steady State Volume of Distribution (L/kg) | 2 |
|  | Plasma Protein Binding (%) | 95.8 |
| Metabolism | CYP1A2 Inhibitor | 0.49 |
|  | CYP2C19 Inhibitor | 0.24 |
|  | CYP2C9 Inhibitor | 0.04 |
|  | CYP2D6 Inhibitor | < 0.01 |
|  | CYP3A4 Inhibitor | 0.36 |
|  | CYP1A2 Substrate | 0.62 |
|  | CYP2C19 Substrate | 0.65 |
|  | CYP2D6 Substrate | 0.41 |
|  | CYP2C9 Substrate | 0.06 |
|  | CYP3A4 Substrate | 0.65 |
| Excretion | Total Clearance (mL/min/kg) | 7.57 |
|  | Half-life | < 3h |
| Toxicity | AMES Mutagenesis | 0.39 |
|  | Carcinogenesis | 0.07 |
|  | Liver Injury (DILI) | 0.29 |
